# Cross-Platform Omics Prediction procedure: a statistical machine learning framework for wider implementation of precision medicine

**DOI:** 10.1038/s41746-022-00618-5

**Published:** 2022-07-04

**Authors:** Kevin Y. X. Wang, Gulietta M. Pupo, Varsha Tembe, Ellis Patrick, Dario Strbenac, Sarah-Jane Schramm, John F. Thompson, Richard A. Scolyer, Samuel Muller, Garth Tarr, Graham J. Mann, Jean Y. H. Yang

**Affiliations:** 1 Charles Perkins Centre, The University of Sydney, Sydney, NSW 2006 Australia; 2 School of Mathematics and Statistics, The University of Sydney, Sydney, NSW 2006 Australia; 3 The Westmead Institute for Medical Research, The University of Sydney, Sydney, NSW 2006 Australia; 4 Melanoma Institute Australia, The University of Sydney, North Sydney, NSW 2006 Australia; 5 Laboratory of Data Discovery for Health Limited (D²4H) Science Park, Hong Kong, SAR China; 6 Department of Melanoma and Surgical Oncology, Royal Prince Alfred Hospital, Sydney, NSW 2050 Australia; 7 Faculty of Medicine and Health, The University of Sydney, Sydney, NSW 2006 Australia; 8 Tissue Pathology and Diagnostic Oncology, Royal Prince Alfred Hospital and NSW Health Pathology, Sydney, NSW 2050 Australia; 9 School of Mathematical and Physical Sciences, Macquarie University, Sydney, NSW 2109 Australia; 10 John Curtin School of Medical Research, Australian National University, Canberra, ACT 2601 Australia

**Keywords:** Prognostic markers, Statistical methods, Melanoma, Machine learning

## Abstract

In this modern era of precision medicine, molecular signatures identified from advanced omics technologies hold great promise to better guide clinical decisions. However, current approaches are often location-specific due to the inherent differences between platforms and across multiple centres, thus limiting the transferability of molecular signatures. We present Cross-Platform Omics Prediction (CPOP), a penalised regression model that can use omics data to predict patient outcomes in a platform-independent manner and across time and experiments. CPOP improves on the traditional prediction framework of using gene-based features by selecting ratio-based features with similar estimated effect sizes. These components gave CPOP the ability to have a stable performance across datasets of similar biology, minimising the effect of technical noise often generated by omics platforms. We present a comprehensive evaluation using melanoma transcriptomics data to demonstrate its potential to be used as a critical part of a clinical screening framework for precision medicine. Additional assessment of generalisation was demonstrated with ovarian cancer and inflammatory bowel disease studies.

## Introduction

New “omics” technologies have allowed the measurement of a wide range of high-throughput molecular information for individual patients over the last 15 years. These high-resolution molecular snapshots hold great promise in today’s era of precision medicine, in which an individual’s molecular measurements can be used to diagnose disease, direct care, guide therapy, and better support patient and clinician decision-making, thus improving over conventional protocols. Despite the large body of literature on published “molecular signatures”, with a few notable exceptions, many of these signatures have limited reproducibility in independent datasets and critical challenges still exist for the effective clinical deployment of omics signatures. Using melanoma cancer as an example, it is apparent that while recent progress in molecular biomarker discovery has found various signatures^1–6^ and have shown strong associations of mRNA expression phenotypes with prognosis in stage III melanoma patients^7^, no validated molecular signature of prognosis is currently endorsed by clinical guidelines for melanoma care, and the few that are commercially available for early-stage melanoma are limited to validating at a single location by the supplier^8^.

Widespread adoption of predictive models could be achieved if omics signatures were measured consistently across different platforms, e.g. when measuring gene expression using anything from microarray to RNA-sequencing. We refer to the ability to extract reliable predictions across heterogeneous samples as “model transferability”, extending the notion of “platform-independent markers”, which was recently explored in selected diseases^9,10^. Here, we describe not only the reproducibility of discovered biomarkers but also the importance of having a model that requires no feature scale adjustment for any incoming new data. In practice, a successful risk model must overcome the challenge of transferability:^11^ it must be able to make predictions on future data without having to re-normalise the data nor to re-train the model. Most omics measurements exhibit high variation between datasets due to batch effects, intra-platform protocol changes, or other site-specific factors; this makes the prediction challenge much harder than when using clinical variables that have clearly defined measurement units such as age, height and weight. This extra “noise” affects the risk model parameters constructed from the training data and creates inaccurate predictions for new and independent data.

Here, we address the transferability challenge for practical implementation by developing a workflow for identifying features that are comparable across multiple datasets and multiple platforms, and for traditional microarray to modern sequencing data. Our Cross-Platform Omics Prediction (CPOP) procedure is a new end-to-end framework that accurately predicts clinical outcomes. We developed CPOP in the context of identifying a gene expression signature that can predict prognosis outcomes in stage III melanoma across different platforms. CPOP meets the transferability challenge through three distinct innovations. First, ratio-based features are constructed, resulting in features that are robust to inter-platform scale differences. Second, the feature selection process incorporates weights that measure the stability of features across multiple datasets. Third, selecting features that have consistent estimated effects across multiple datasets in the presence of noise. The model constructed for stage III melanoma is validated on two published melanoma studies^12,13^ and a newly generated dataset (*n* = 46) based on an independent cohort. Furthermore, CPOP’s utility is further illustrated on two other complex diseases, ovarian cancer and inflammatory bowel disease (IBD).

An interactive web portal (http://shiny.maths.usyd.edu.au/CPOP/) has been constructed to illustrate how gene expression data can be uploaded and predictions of outcomes for stage III melanoma patients can be obtained in real-time. Together, these innovations and methodologies deliver a molecular risk prediction platform with statistical modelling improvements, thereby producing a coherent framework that can be adopted in multi-centre and prospective settings.

## Results

### Cross-Platform Omics Prediction (CPOP), a robust procedure to select transferable biomarkers

**Fig. 1:**
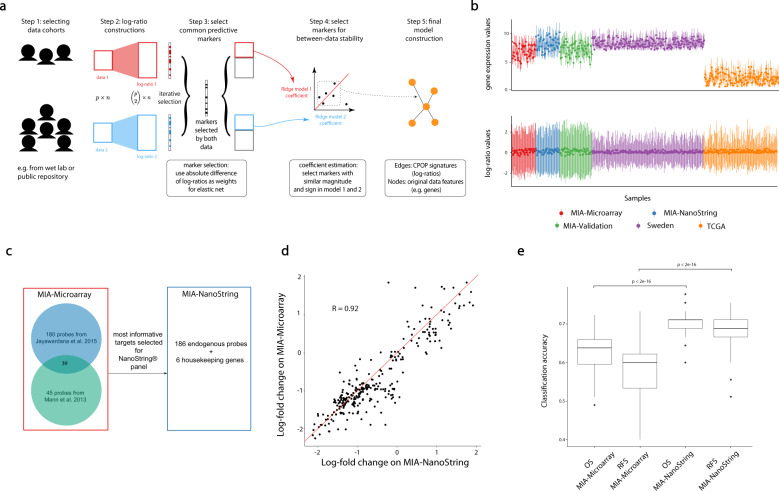
Overview of CPOP and the motivating melanoma dataset. **a** Schematic illustration of the five-step CPOP procedure with emphasis on the stable selection of features in Steps 3 and 4. **b** Quartile plot of the expression values of all genes (top panel) and all pairwise (bottom panel) log-ratio features for each sample (*n* = 488) in the melanoma data collection. Each sample is represented by the median (a single solid point), the first quartile (the lower end of a vertical line) and the third quartile (the upper end of a vertical line) of all the gene/feature values for that sample. **c** NanoString probe selection (186 probes) based on results from our previous microarray studies^7,14^. **d** Scatter plot of log fold-change for genes common between MIA-Microarray and MIA-NanoString. **e** Boxplot comparisons of overall accuracy for overall survival (OS) and recurrence-free survival (RFS) between the MIA-Microarray and MIA-NanoString data. The y-axis shows the classification accuracy calculated from 100 repeated 5-fold cross-validation using a support vector machine classifier. The good/poor prognosis classes for overall survival (OS) and recurrence-free survival (RFS) are defined in Methods. The centreline of a boxplot denotes the median classification accuracy, and the lower and upper bounds of the box denote the first and third quartile values, respectively. The lower and upper whiskers denote 1.5 times the interquartile range away from the first and third quartile values, respectively.

To address challenges associated with the practical implementation of the risk models, we developed a robust Cross-Platform Omics Prediction (CPOP) procedure, a five-step approach to select transferable biomarkers (Fig. 1a, see Methods and Supplementary Materials for a detailed description). The CPOP procedure begins by identifying a collection of datasets with similar clinical outcomes that can be used for joint modelling. This may entail extracting relevant data from a public repository or developing a clinical-ready molecular assay. Following that, CPOP creates features that are the ratio of each gene’s expression to that of other genes and then identifies from these ratios those that are associated with clinical outcomes. Features that are predictive of the clinical outcome are then selected using a regularised regression modelling framework, a new strategy that focuses on identifying features with consistent effect sizes across multiple datasets. These selected features serve as the markers used for final model construction. Further detail is presented in the Methods section and in the Supplementary Materials.

CPOP overcomes the transferability challenges through the use of three key strategies, which identify consistent features on relative gene-expression differences across many datasets. The collection of melanoma data presented in Fig. 1b (upper panel) illustrates the large location and scale differences between datasets that are typically observed in gene expression data. First, biomarkers are ratio-based values rather than absolute expression levels. Our ratio-based strategy is different from many others that adjust all gene expressions by one or a group of specified control genes. Our method of examining all pairs of features eliminates the requirement for pre-determined sets of control genes and accurately captures the relative changes in a gene expression system. As a consequence, the use of ratio-based gene expression features reduces between-data variation as shown in Fig. 1b (lower panel). Second, each feature is assigned a weight proportional to its between-data stability, in contrast to traditional modelling procedures where only a single homogeneous data is used for modelling. Third, CPOP selects features that yield consistent estimated effects in the presence of between-data noise, thus strengthening reproducibility across datasets containing similar biological signals. The conceptual innovation here is to identify signals that are consistent in the presence of unwanted variation rather than removing unwanted variation. Our CPOP procedure was developed for and is applicable to any type of outcome, whether diagnostic, prognostic or predictive of treatment response.

### Designed clinical-ready assay platform to deploy extended gene signature

Informative datasets for the CPOP procedure can either be identified in public literature or constructed from experiments. Here, we constructed a clinical-ready molecular assay using the NanoString nCounter™ platform (NanoString Technologies Inc.). This technology was chosen for its low per-assay cost and wide deployment. We constructed a gene set panel (Fig. 1c) consisting of 186 differentially expressed (DE) genes that were most strongly associated with prognosis in our previous microarray study cohort^7,14^, known as the Melanoma Institute Australia discovery (MIA discovery) cohort, and six housekeeping genes. The full list of probes is presented in Supplementary Table 3. The baseline characteristics of the MIA discovery cohort are described in Table 1, and Supplementary Fig. 1 shows the survival time distribution of the individuals. Next, we generated gene expression data from the MIA discovery cohort (*n* = 45) using the new NanoString platform and the resulting dataset is referred to as “MIA-NanoString”.

**Table 1:** Data summaries for five melanoma datasets, MIA-Microarray, MIA-NanoString, TCGA, Sweden and MIA-Validation.

	MIA-Microarray and MIA-NanoString	TCGA	Sweden	MIA-validation
Number of samples	45	139	210	46
Median age (years)	62	56	64	61
Sex F	17 (38%)	59 (42%)	86 (41%)	14 (30%)
*M*	28 (62%)	80 (58%)	124 (59%)	32 (70%)
Stage/metastasis type	Stage III: 45 (100%)	Stage III: 139 (100%)	General: 23 (11%)	Stage III: 46 (100%)
			In-transit: 15 (7%)	
			Local: 11 (5%)	
			Primary: 15 (7%)	
			Regional:139 (66%)	
			NA: 7 (3%)	
Median survival (months)	OS: 22	OS: 26.9	DSS: 17.6	OS: 65.5
	RFS: 8			RFS: 9
Survival status (Alive)	Yes: 19 (42%)	Yes: 78 (56%)	Yes: 108 (51%)	Yes: 26 (57%)
	No: 26 (58%)	No: 61 (44%)	No: 102 (49%)	No: 20 (43%)

Included are the number of samples, the median age of the cohort, gender (sex), the median survival time in month and survival status. OS, RFS and DSS refer to overall survival, recurrence-free survival and disease-specific survival, respectively.

A direct comparison of the MIA-NanoString data to the previously generated data using the Illumina cDNA microarray platform (referred to as “MIA-Microarray”) on the same MIA discovery cohort shows similar analytical results. We confirmed that both the gene expression (Supplementary Fig. 2) and the log-fold-differences of gene expression values between the good and poor prognosis groups measured by the new NanoString assay are very highly correlated (*r* = 0.9) with those originally measured in MIA-Microarray (Fig. 1d). Based on the recurrence-free survival (RFS), overall survival (OS) and the survival status of the patients, we grouped patients into different prognosis groups and confirmed that the classification performance of matching patients using the new NanoString assay is better than the original cDNA microarray data (Fig. 1e).

### CPOP shows improved transferable performance and robustness compared to competing procedure

**Fig. 2:**
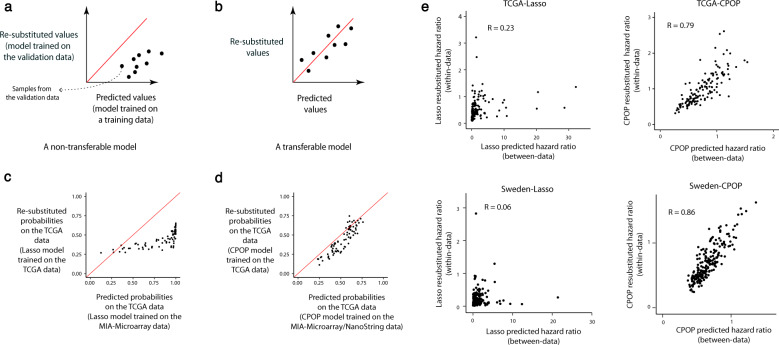
Schematic drawings and figures for the comparison between prediction values and re-substituted values. **a**, **b** Schematic drawing of a non-transferable and a transferable model, respectively. By fixing the samples in a validation data to be the same, we can compare a model’s prediction values (a model trained using training samples independent of the validation data) and the re-substituted predicted value (a model with the same configurations but trained using the validation data itself). A transferable model should produce predicted values on identical scales as the re-substituted predicted values and so each point on the scatterplot, representing a sample in the validation data, should be randomly scattered around the identity line (y = x). A non-transferable model typically exhibits biases and clustering away from the identity line. **c** A real-data illustration of a non-transferable Lasso model, trained on the MIA-Microarray data and validated on the TCGA melanoma data. **d** A real-data illustration of a transferable CPOP model, trained on the MIA-Microarray and MIA-NanoString data and validated on the TCGA melanoma data. **e** Scatter plot illustrating the concordance between between-data predicted hazard ratios and within-data (re-substituted) hazard ratios. We trained a CPOP penalised Cox model with recurrence-free survival times as the response by combining the MIA-Microarray and MIA-NanoString data. Each of the four panels illustrates a combination of the Lasso and the CPOP model predictions on the TCGA and the Sweden data. R denotes the Pearson’s correlation coefficient.

We apply the CPOP procedure to the MIA-Microarray and MIA-NanoString data to identify a molecular signature and construct a corresponding prognostic model. We demonstrate transferable properties as shown in the schematic drawing in Fig. 2a, b. We construct a model using one dataset, A, and apply the model to a new dataset, B, to generate a prediction outcome. This “cross-data predicted outcome” (*x*-axis) reflects the prediction outcome across platforms without renormalisation. We compare the prediction findings to an ideal situation with no scale differences, where we build a model from dataset B and apply it again to dataset B. We refer to this result as “within-data prediction outcome” (*y*-axis). A transferable model (Fig. 2b) will produce approximately the same cross-data prediction outcome (*x*-axis) as the within-data prediction outcome (*y*-axis). That is, on a scatter plot we expect the results from these two models to cluster around the identity line (i.e. y = x).

Here, we applied the CPOP procedure to generate a transferable model using the MIA-Microarray and MIA-NanoString datasets and applied the model to the TCGA melanoma data. Figure 2c shows predicted probabilities built using the Lasso regression resulting in a scale difference between the cross-platform and within-platform predicted outcome with the TCGA data (similar evaluation is also presented in Supplementary Fig. 3). In contrast, Fig. 2d shows that CPOP produces predicted probabilities that are essentially the same as the “desired” within-data prediction outcome built. Next, we examined the concordance of the between-data prediction hazard ratios and within-data (re-substituted) hazard ratios. We applied the CPOP procedure to generate a transferable model for survival times using the MIA-Microarray and MIA-NanoString data and used the model to make predictions on the TCGA and the Sweden datasets. The panels in Fig. 2e compare the hazard ratio prediction under the between-data setting (x-axis, with MIA-NanoString and MIA-Microarray data as the training data) and the idealistic within-data setting (y-axis, with either TCGA or Sweden data as the validation data) and a transferable model will show a high correlation of hazard ratio estimated in these two settings (see Methods). Here, we demonstrate CPOP’s ability to produce predictions that are on an equal scale and show major improvement (substantially higher correlation; 0.79 vs 0.23 in TCGA and 0.86 vs 0.06 in Sweden) compared to the commonly used Lasso regression^15^ (Supplementary Fig. 4). The results of both of these analyses indicate the stability of the model’s coefficients in a between-data or cross-platform setting.

We next illustrate the robustness of the CPOP model to missing features from incoming data or new samples. This type of missingness is a common challenge arising in the event of a failure of some probes (e.g. due to reagent or laboratory error) with some features entirely not captured. Such a situation with entire missing features is unlike in classical missing value problems where the missingness occurs in the training data. Here, we examine the situation where we have missingness in the incoming sample. CPOP handles this situation by using additional regression models on the reference training data to impute the missing feature in the new/test data. We created two simulations and show our model is robust to this type of missing data (Supplementary Figs. 5 and 6). This makes the final implemented CPOP highly suitable for the assimilation of gene expression data from diverse platforms and cohorts.

### CPOP validates well in external independent melanoma cohorts without further normalisation or model modification

**Fig. 3:**
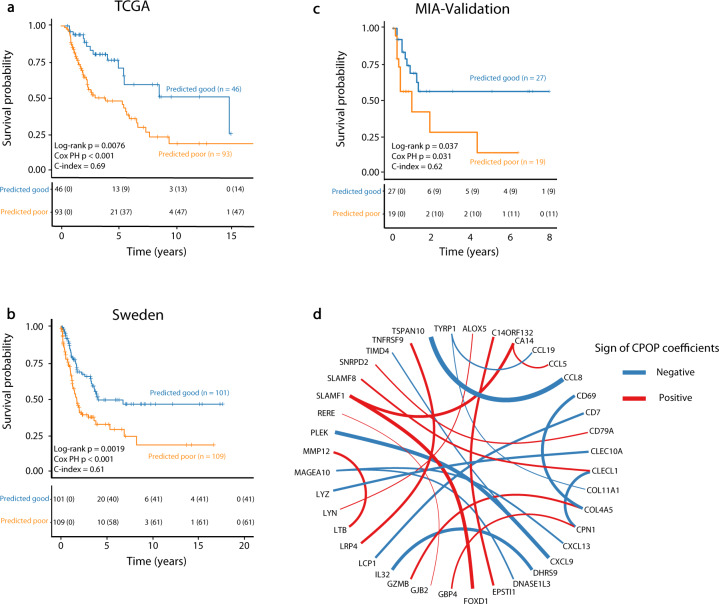
Validation results of the melanoma dataset. **a** Kaplan–Meier plots show a significant difference in survival probability between the predicted good (blue line) and poor (orange line) prognostic classes on the TCGA (*n* = 139). The CPOP model here is trained on MIA-Microarray and MIA-NanoString based on the RFS-defined prognosis classes. **b** Similar evaluation for the Sweden (*n* = 210) data. **c** Similar evaluation for the MIA-Validation data (*n* = 46) (including four imputed genes). **d** Network visualisation of the final CPOP model highlights the ratio-based signatures developed from applying the CPOP model on the MIA-Microarray and MIA-NanoString data. Each node of the network represents a gene and an edge connecting two genes (nodes) represents the log-ratio feature that is present in the signature. The colour and thickness of each edge represent the sign and the magnitude of the size of the estimated coefficients, respectively. The genes are in alpha-numeric ordering.

To further assess “model transferability” of the CPOP model on external data, we applied the trained melanoma model on the TCGA^12^ and Sweden^13^ data without further data normalisation or model modification and examined its ability in predicting samples of different survival classes. The Kaplan–Meier^16^ (KM) plots show significant differences in survival probability between the good and poor predicted classes from the CPOP model in TCGA (Fig. 3a, *p* = 0.0076) and in the Sweden data (Fig. 3b, *p* = 0.0019). In contrast, KM-plots that were generated for the Lasso model in Supplementary Fig. 7 did not show statistically significant difference (*p* = 0.096 for TCGA and *p* = 0.062 for the Sweden data). As the training data (MIA-Microarray and MIA-NanoString) and the validation data (TCGA and Sweden) were generated from distinct gene expression platforms and distinct samples, we have demonstrated that the CPOP procedure is able to select features that are stable between platforms and sample selection. We then establish the robustness of our results by repeating the cross-data validation using different training-testing pairs. Supplementary Fig. 8 illustrates that 19 (79%) out of 24 combinations of training-testing pairs in the melanoma data collection show statistical significance with *p* < 0.05. Furthermore, Supplementary Fig. 9 show that the CPOP procedure with non-normalised log-ratio features can have an equal or improved performance compared to the Lasso model with ComBat^17^ normalisation on the log-ratio features.

The CPOP procedure identified the most stable relationships between gene expression values in diverse datasets, which may enhance biological interpretation. The CPOP melanoma signature is based on relative expression levels between genes and after applying the CPOP procedure onto the MIA-Microarray and MIA-NanoString data, the final CPOP signature consisted of 24 log-ratio features derived from 40 genes as presented in Fig. 3d and Supplementary Table 4. In this figure, an edge connecting two genes (nodes) indicates the ratio is present in the CPOP signature and its thickness indicates the magnitude of coefficient values in the predictive model and their direction of correlation, respectively. Genes most strongly implicated include CXCL9^18,19^, CCL8^20^, TSPAN10, PLEK, CLEC10A^21^, SLAMF1, CLECL1, MAGEA10, CXCL13 and LYZ^22^, which all have been identified as potential biomarkers in previous studies and show enrichment in the inflammatory response.

### New independent validation cohort confirms CPOP’s suitability for prospective study

As a final validation of the CPOP-selected melanoma signature, we further generated an independent cohort (referred to as “MIA-Validation”, *n* = 46, see Methods) in 2020. These samples were obtained from Melanoma Institute Australia and were accrued since 2010 at the same centre, as described in Table 1 and Supplementary Table 1. These samples served as an independent and prospective validation of our methodology. We applied our CPOP model to this cohort and this gives a significant difference in survival probability in the KM plot between the predicted good and poor predicted classes in Fig. 3c (*p* = 0.037). CPOP demonstrated a consistent performance (evaluation framework shown in Supplementary Fig. 10) in transcriptome data from different gene expression platforms (microarray, RNA-Seq and NanoString), and in independent cohorts widely separated in time and space. This further validates the CPOP procedure and its suitability for prospective settings and potential for wide-scale deployment for clinical prognosis.

### The generalisability of the CPOP procedure is illustrated in two additional diseases

The need for model transferability and applications of the CPOP procedure is presented in another two case studies. In the first case study of ovarian cancer, we examined 1,189 samples collected across nine different studies^23^ that are varied in clinical survival times (Supplementary Fig. 11). The heterogeneity of study cohorts presented a unique challenge that was only previously dealt with using meta-analysis or pooling and reprocessing. We applied CPOP from two selected studies^24,25^ to identify and build a transferable signature and model. We were then able to make stable cross-study predictions on the remaining seven datasets (Supplementary Fig. 12), further highlighting the transferability of the CPOP signature and risk model.

**Fig. 4:**
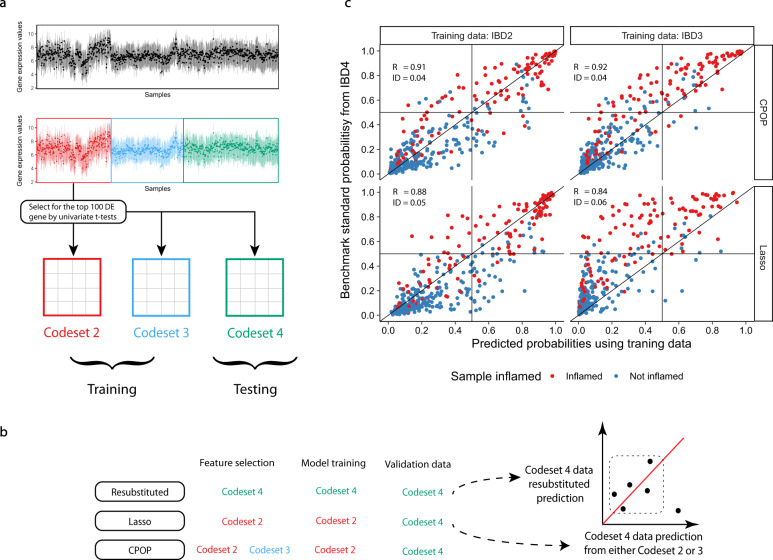
Validation results of the IBD dataset. **a** Schematic drawing of the pre-processing steps of the IBD data. Due to reagent set changes, samples in this data are separated into three major batches. Top 100 DE genes are selected from the IBD2 cohort to reduce the number of features used for modelling. **b** Schematic drawing of the evaluation steps of the IBD data. Treating both the CPOP and Lasso methods as feature selection methods only and using the ridge regression model, we compare the predicted values against benchmark standard values where the feature selection, model training and validation data are identical. **c** Scatter plots comparing predicted probabilities of inflammation. The CPOP method is able to produce prediction values that are more consistent between different batches (codesets) with higher correlation (denoted as R in the figure) and closer to the benchmark standard prediction values (denoted as ID in the figure).

In another case study of inflammatory bowel disease^26^, a total of 983 samples were assayed on a customised NanoString panel in three distinct batches across two years, see Fig. 4a and Supplementary Fig. 13. Such a batch effect is typical of a prospective study utilising omics technologies over an extended period of time. Here, we apply CPOP to account for the change between distinct batches by training our model on the first two batches and see if we can predict the outcome without renormalisation. Figure 4b presents our evaluation strategy for the feature selection property of the CPOP method where the more stable methods will have points more tightly correlated on the y = x line on the scatter plot. Figure 4c shows that CPOP again outperforms Lasso regression in concordance performance metrics and is able to make a more stable cross-batch prediction on inflammation samples demonstrating its strength for large cohort prospective study.

## Discussion

This paper presents a new transferable procedure, CPOP, for biomarker omic analysis motivated by challenges in melanoma research. To the best of our knowledge, this is the first transferable risk prediction model for melanoma with a strong emphasis on addressing practical challenges in the implementation of biomarkers/assays.

Importantly, the concept of transferability is not the same as data integration, data harmonisation or data normalisation. To date, the most popular method for adjusting data scales between different datasets is through the use of normalisation, often divided into two broad categories of z-score standardisation and between-data normalisation. We refer to z-score standardisation as a pre-processing step where each omics predictor is centred at zero and its sample variance is scaled to one. This procedure has been widely used, for example for investigating ovarian cancer^23^. On the other hand, between-data normalisation aims to correct the statistical distributions of genes in the validation data to be similar to that of training data. Through the act of combining multiple data and calculating summary statistics like a sample-wise mean, both of these methods introduce interdependencies between the samples and may not be suitable for processing and predicting on a single omics profile (Criterion 15 in McShane et al.^27^). Here, we are addressing a translational gap where renormalisation for incoming samples together with an existing cohort is not possible due to constraints with consent, data security, or other practical reasons. Therefore, data normalisation alone is not enough to address the various challenges in implementing precision medicine utilising omics data with additional considerations relating to the model transferability and reproducibility also being necessary.

It is important to highlight that while the calculation of (log)-ratios is not a new idea, the work presented in this paper challenges the practice of using only genes as features but not functions of sets of genes. Specifically, in our framework, we propose using (log-)ratios of all gene pairs as potential features when working with transcriptomics data. Traditionally, most model prediction frameworks begin with value adjustments on genes through the use of either endogenous or artificial “control genes”. However, in practice, it often means that the values of these control genes become dominant in deriving any prediction score, sometimes even more dominant than the statistical prediction model itself. This is problematic especially given the controls can be dataset-dependent. The calculation of these (log-)ratios is intended to obtain a quantitative measure for the relative differences between the gene expression values, which we have found to be better preserved across different patient cohorts and data generation platforms. As this (log-)ratio value is calculated within a sample, we are assuming that the relative interactions between the omics features are dataset-independent and cross-platform, provided that all the omics platforms in question can unbiasedly estimate the relative expression level of features. The prefix of ‘log’ simply reflects the prevalence of the log transformation in dealing with omics data. While this all-pairwise feature construction is not suitable to be applied to the whole genome (e.g. whole genome RNA-sequencing) directly due to the large dimensionality, this approach is achievable on targeted omics assays. These targeted omics assays typically provide a higher signal-to-noise ratio for candidate features that are of higher clinical relevance and are in wide use in clinical validation, translational work and in the implementation phase of precision medicine. It is critical to note that this concept of ‘relative difference’ is not limited to transcriptomics (e.g. similar attempts were made in Qin et al.^28^).

The CPOP procedure combines ratio-feature construction and using penalised regression classifiers. A natural question is to ask if the regression method under the CPOP procedure can be replaced by another state-of-the-art method, e.g. random forest, transfer learning or neural network. We need to first acknowledge that any machine learning method does not automatically resolve feature scaling differences. Because the CPOP procedure is intended to provide interpretability and transparency in feature selection, an ensemble-type model (e.g. random forest) that does not state explicitly how decisions are made is not considered as such a model would obscure clinical interpretability. We ultimately opted for a regression-based modelling approach because of its relative simplicity and prevalence in the literature. In the CPOP procedure, the difference in the scaling of features is further mitigated by using weights in the penalised regression model to preferentially select features with stable distributions and estimates across multiple datasets. While in principle it is possible to derive platform and batch agnostic features or weighted statistics for other machine learning methods as the training sample size increases, the resulting procedure will still need to be carefully adapted and tested to ensure that the increase in model complexity is also matched by the relative gain in accuracy.

In summary, in order to enable multi-centre implementation of gene expression signatures of prognosis in stage III melanoma specifically and omics signatures more widely in clinical practice, we have developed a new Cross-Platform Omics Prediction (CPOP) procedure that closes an important gap between molecular biomarker discovery and wider clinical use. The CPOP approach accounts for differences in feature scaling in omics data by performing weighted feature selection and estimation that preferentially selects stable features across multiple datasets. Our application of the CPOP method on multiple melanoma studies and validation work using the NanoString technology serve as important components towards the future implementation of this methodology into clinical practice. We construct a web portal that is available online at http://shiny.maths.usyd.edu.au/CPOP/ for prognostic prediction in stage III melanoma patients. This interactive tool illustrates how gene expression data can be uploaded and predictions to develop stage III melanoma can be obtained in real-time. For creating CPOP models for other data and diseases, an R package is made available at https://sydneybiox.github.io/CPOP/. Using the CPOP package, we demonstrate the generalisability of the procedure to two other diseases: an ovarian cancer data collection (nine datasets) and a large IBD dataset with 983 samples. In conclusion, we deliver a molecular (omics) risk prediction platform with substantial improvements in reproducibility and stability that can be adopted in multi-centre and prospective settings.

## Methods

### Melanoma molecular signature assay with the NanoString nCounter platform

#### NanoString sample selection

Tumour samples were obtained from the Melanoma Institute Australia (MIA) Biospecimen Bank, a prospective collection of fresh-frozen tumours accrued with written informed patient consent and Institutional Review Board approval (Sydney South West Area Health Service institutional ethics review committee (Royal Prince Alfred Hospital (RPAH) Zone) Protocol No. X08-0155/HREC 08/RPAH/262, No. X11-0023/HREC 11/RPAH/32, and No. X07-0202/HREC/07/RPAH/30) since 1996 through MIA, formerly the Sydney Melanoma Unit. Both the data, MIA-Nanostring and MIA-Validation were generated (see ‘Melanoma data collection’ section below for details) from this biospecimen bank with non-intersecting samples.

#### NanoString assay construction

Gene expression profiling was carried out using the NanoString nCounter® platform (Seattle, WA). NanoString designed and manufactured customised probes corresponding to 192 probes. 186 of these were identified in our previously reported studies^7,14^. Of the 186 probes, 46 were found to be differentially expressed genes between poor and good prognosis patients (stratified based on overall survival time and vital status^7^), and 180 probes were identified in a model built to predict prognosis^7^. Between the two studies, 39 of these probes overlapped. In instances where there was more than one probe per gene, the most informative probe based on an inclusion frequency of 30% for that particular gene was included in the NanoString panel. Six housekeeping genes were selected from a list of previously reported housekeeping genes^29^ and had variances in the lowest quintile (20%) of the data presented in two previous studies^7,14^, a differential expression *p* value greater than 0.5. These housekeeping genes were selected to cover a range of high expression (CENPB, CTBP1, GNB2L1), medium (RERE, SNRPD2) and low expression (UQCR). A complete list of the customised probe set is given in Supplementary Table 3.

#### NanoString experimental procedure

Tumour RNA was extracted as previously described by Mann et al.^7^ RNA purity and concentration were assessed using the Agilent TapeStation system. The nCounter® gene expression assay (NanoString Technologies, Seattle, WA) was performed according to the manufacturer’s instructions using 100 ng of total RNA. For each assay, a high-density scan (encompassing 600 fields of view) was performed.

#### NanoString hybridisation protocol

A thermal cycler is preheated to 67 °C. Tagset and RNA samples are removed from a freezer and thawed at room temperature. The tubes are inverted to mix and then briefly spun down. A hybridisation master mix is created by adding 70 μL of hybridisation buffer and 7 μL of the Probe A working pool directly to the tube containing the Tagset. The solution is inverted several times to mix and spin down. 7 μL of the Probe B working pool is added to the master mix. 8 μL of the master mix is added to each of the 12 strip tubes then 7 μL of RNA sample is added to each tube. Strip tubes are inverted several times and flicked to increase mixing. The tubes are briefly spun and immediately placed in the preheated 67 °C thermal cycler, incubated for 16 h and then ramped down to 4 °C.

#### NanoString data pre-processing and quality assessment

NanoString data was read into R^30^ using the *NanoStringQCPro* package^31^. For the purpose of illustrating cross-platform noise, we performed a simple log2-transformation on the raw counts. This allows us to assess if the panel can facilitate prospective experiments without model modification. Of all the samples measured in the “MIA-NanoString” data, 45 samples overlap with previous microarray studies^7,14^, named “MIA-Microarray”. This allows us to correlate genes between the two data in Supplementary Fig. 2. Of the 192 common genes (matched through official gene symbols), the median correlation is 0.86 with the first and third quartiles being 0.79 and 0.90 respectively.

#### Statistical analysis - RFS assessment

Clinical follow-up time for samples presented in Mann et al.^7^ and Jayawardana et al.^14^ is updated in 2018 and we define recurrence-free survival (RFS) as the time difference between the date of the first recurrence after tissue banking and the date of tumour banked. Based on RFS, we further defined:

- “Good” prognosis group being RFS greater than four years and alive with no sign of recurrence.
- “Poor” prognosis group being RFS less than one year and died due to melanoma.

This resulted in 19 samples in the good prognosis group and 26 samples in the poor prognosis group. Using these RFS-defined prognosis classes for these 45 samples, we use limma^32^ to compute moderated *t*-statistics for all 192 overlapped genes.

#### Statistical analysis - Prognostic assessment

We validate the prognostic assessment for the MIA-NanoString datasets by comparing its performance accuracy for both overall survival (OS) and RFS against the same samples in the MIA-Microarray data. Performance accuracy is obtained by using the *ClassifyR*^33^ package to compute 100 repeats of 5-fold cross-validation with the limma^32^ feature selection and support vector machine classifier. This is specified via the parameters *ResubstituteParams(nFeatures* = *c(20, 50, 100), performanceType* = *“balanced error”, better* = *“lower”)* before executing the function *runTests*. All analyses are performed in R^30^ version 3.6.2.

### Data collection

- Melanoma data collection: We collected five melanoma datasets mostly consisting of late-stage samples, see Supplementary Table 1. Three of these data are samples from the MIA and the other two are publicly available data. Of the three MIA data, one is measured by Illumina microarray technology and two are measured by our customised NanoString assay as described in the “Melanoma molecular signature assay with the NanoString nCounter platform” section.
- Ovarian cancer data collection: Data is obtained from the curatedOvarianCancer Bioconductor package and analysed using the process described by Waldron et al.^23^ and Yoshihara et al.^24^, with a few modifications (detailed in Supplementary Information). We focus on the 126-genes signature reported in Yoshihara et al.^24^ and select genes that are also present in all nine datasets (Supplementary Table 2). This results in 94 genes with corresponding 4371 log-ratio features.
- Inflammatory bowel disease data: A study of the inflammatory bowel disease (IBD) with 983 samples and 712 genes. Raw NanoString data is downloaded from Gene Expression Omnibus with accession GSE73094. The data contains three batches, IBD2 (*n* = 303), IBD3 (*n* = 295) and IBD4 (*n* = 385) from chemical reagent changes^26^.

Detailed descriptions of the processing workflow for all three data collections are provided in Supplementary Materials.

### Cross-Platform Omics Prediction (CPOP) method overview

Cross-Platform Omics Prediction (CPOP) is a procedure that enables sample prediction across gene expression datasets with different scales (e.g. different sample means). We will use the generic phrase of “scale difference” to encompass all situations where multiple gene expression data exhibit different scales in the data due to, for example, the use of different experimental instruments/platforms or drifts in measurements in a prospective setting. We use the term “biomarker” and “feature” interchangeably. We will use the term *predictive* in a statistical sense and the term *predictive markers* in a generic way, referring to all forms of biomarkers, whether they are diagnostic, prognostic or predictive. We use the term *training set* interchangeably with *reference set* (or *sets*), and restrict usage of the term *test set* or *validation set* to situations with known patient outcomes, i.e. to situations where we are assessing or comparing the performance of CPOP. We use the term *test sample* or *validation sample* when the unknown subjects are to be predicted.

A major consideration in developing CPOP is to make predictions on a single sample without normalisation or combining it with additional data. The CPOP procedure has the following three key characteristics:

1. CPOP uses (log)-ratios of genes as biomarkers (features), which are more stable than using individual gene expression values (Step 2 of CPOP).
2. CPOP uses the Elastic Net model^34^ to perform feature selection using weights proportional to the stability of features across more than one dataset. This allows the selection of common predictive markers (Step 3 of CPOP).
3. CPOP selects for features with high similarity in their between-data estimated effects (Step 4 of CPOP).

Suppose we have an omics data matrix, ***X***, of size *n* × *p*, where *n* is the number of samples and *p* is the number of omics features (e.g. genes on a gene expression platform). We define the “log-ratio matrix” as a matrix, ***Z***, of dimension *n* × *q*, where $$q = \left( {\begin{array}{*{20}{c}} p \\ 2 \end{array}} \right)$$ and each column of ***Z*** is the pairwise difference between two log-transformed columns in ***X***. Formally, each column of ***Z*** is given by enumerating all log-ratio features ${{{\mathrm{log}}}}\left( {x_{{{\mathrm{l}}}}} \right) - \log \left( {x_{{{\mathrm{m}}}}} \right)$ for $1 \le l\, < \,m \le q$, where *x*_*l*_ and *x*_*m*_ being columns of **X**.

In the Main Fig. 1a, CPOP is presented as a five-step procedure. These steps can be further described as follow.

1. Data selection: the first step of data selection is dependent on the research questions to be addressed and one should select data with similar and appropriate clinical outcomes of interest. For example, the selected cohort can consist of independent samples at the same cancer stage. In the rest of the procedure, we assume we have two gene expression data and the CPOP model training will aim to find features consistently predictive in both data.
2. Log-ratio matrices construction: The associated log-ratio matrices for the two gene expression data are constructed as above and denoted as ***Z***_1_ with size *n*_1_ × *q* and ***Z***_2_ with size *n*_2_ × *q*. Here, *n*_1_ and *n*_2_ are the sample sizes for the two datasets, respectively. We do not impose the restriction of paired samples across the two data; however, we assume the two data measure the same *q* log-ratio features, or we restrict our modelling to the common *q* log-ratio features between the two datasets. For both data, we also have a clinical outcome measurement, denoted as *y*_1_ and *y*_2_ associated with data 1 and 2 respectively.
3. Selecting common predictive features: Fit a Weighted Elastic Net (WEN) model (see Supplementary Materials for more details) for both datasets ***Z***_1_ and ***Z***_2_ to obtain estimated regression coefficients ${{{\hat{\boldsymbol \beta }}}}_1^{\left( 1 \right)}$ and ${{{\hat{\boldsymbol \beta }}}}_2^{\left( 1 \right)}$ for datasets ***Z***_1_ and ***Z***_2_ respectively. The superscript denotes that this is the *first* step of the CPOP feature selection. In fitting the WEN models, we propose to use weights that measure the similarities between log-ratio features in the two data. In this paper, we primarily use the differences between column-means as weights: $w_j = \left| {{{{\mathrm{mean}}}}\left( {{{{\boldsymbol{Z}}}}_{1j}} \right) - {{{\mathrm{mean}}}}\left( {{{{\boldsymbol{Z}}}}_{2j}} \right)} \right|$ for each $j = 1, \ldots ,q$. Other choices for weights can be specified by the end-users in our CPOP package. Since WEN generates sparse estimates, thus it also naturally selects features from our data as those features with non-zero estimates in ${{{\hat{\boldsymbol \beta }}}}_1^{\left( 1 \right)}$ and ${{{\hat{\boldsymbol \beta }}}}_2^{\left( 1 \right)}$. We further define a set of features that collects all non-zero features selected into both models in both data. Mathematically, this set can be written as $S^{\left( 1 \right)} = \left\{ {j\left| {{{{\hat{\boldsymbol \beta }}}}_{1,j}^{\left( 1 \right)} \,\ne\, 0;{{{\hat{\boldsymbol \beta }}}}_{2,j}^{\left( 1 \right)} \,\ne\, 0} \right.} \right\}$. An iterative component can be added to this step to enhance the quality of feature selection, see Supplementary Materials.
4. Selecting features with between-data stability: Next, we fit an unweighted ridge regression model to the two matrices including only features present in *S*^(1)^ and obtain ${{{\hat{\boldsymbol \beta }}}}_1^{\left( 2 \right)}$ and ${{{\hat{\boldsymbol \beta }}}}_2^{\left( 2 \right)}$. This is the *second* feature selection step where we select features with coefficients that are similar between both data. Mathematically, this set can be written as $S^{\left( 2 \right)} = \left\{ {j\left| {{{{\mathrm{sign}}}}\left( {{{{\hat{\boldsymbol \beta }}}}_{1,j}^{\left( 2 \right)}} \right) = {{{\mathrm{sign}}}}\left( {{{{\hat{\boldsymbol \beta }}}}_{2,j}^{\left( 2 \right)}} \right)} \right.} \right\}$. An iterative component can be added to this step to enhance the quality of feature selection, see Supplementary Materials.
5. Final model estimation: The final CPOP models are the unweighted ridge regression models fitted on the subset of the data including only features present in *S*^(2)^, which are features that are common between two datasets and with similar coefficients. We will refer to these models as ${{{\hat{\boldsymbol \beta }}}}_1^{CPOP}$ and ${{{\hat{\boldsymbol \beta }}}}_2^{CPOP}$, respectively. Predictions on new samples could be made by taking the average of the two to produce a singular ${{{\hat{\boldsymbol \beta }}}}^{CPOP}$.

Additional methodological details, including statements of data collection and curation, mathematical formulations and method evaluations and discussions are available in Supplementary Materials. The CPOP package is available at https://sydneybiox.github.io/CPOP.

### Reporting summary

Further information on research design is available in the Nature Research Reporting Summary linked to this article.

## Supplementary information

Supplementary materials + Supplementary table 1 and 2

Supplementary table 3

Supplementary table 4

Reporting Summary

## Data Availability

The MIA generated Microarray and NanoString data are available at Gene Expression Omnibus (GEO) with the accession ID GSE54467 and GSE156030, respectively. The TCGA melanoma data were downloaded from the TCGA portal and the Sweden data was downloaded from GEO with the ID GSE65904. The inflammatory bowel disease data were downloaded from GEO with the ID GSE73094. The ovarian cancer data was downloaded using the cureatedOvarianCancer Bioconductor package.

## Code availability

R package is available at https://github.com/sydneybiox/CPOP. Customised scripts for generating figures in this manuscript is available at https://github.com/SydneyBioX/CPOP_public.

## References

[1] Amaral TMS (2020). Clinical validation of a prognostic 11-gene expression profiling score in prospectively collected FFPE tissue of patients with AJCC v8 stage II cutaneous melanoma. Eur. J. Cancer.

[2] Diefenbach, R. J. et al. Design and testing of a custom melanoma next generation sequencing panel for analysis of circulating tumor DNA. Cancers 12, 2228. https://www.mdpi.com/2072-6694/12/8/2228 (2020). 10.3390/cancers12082228 PMC7465941 32785074

[3] Greenhaw BN (2020). Molecular risk prediction in cutaneous melanoma: a meta-analysis of the 31-gene expression profile prognostic test in 1,479 patients. J. Am. Acad. Dermatol..

[4] Gambichler T (2021). Prognostic significance of an 11-gene RNA assay in archival tissue of cutaneous melanoma stage I-III patients. Eur. J. Cancer.

[5] Garg M (2021). Tumour gene expression signature in primary melanoma predicts long-term outcomes. Nat. Commun..

[6] Dubin DP, Dinehart SM, Farberg AS (2019). Level of evidence review for a gene expression profile test for cutaneous melanoma. Am. J. Clin. Dermatol..

[7] Mann GJ (2013). BRAF mutation, NRAS mutation, and the absence of an immune-related expressed gene profile predict poor outcome in patients with stage III Melanoma. J. Invest. Dermatol..

[8] Grossman, D. et al. Prognostic gene expression profiling in cutaneous melanoma: identifying the knowledge gaps and assessing the clinical benefit. *JAMA Dermatol*. 10.1001/jamadermatol.2020.1729 (2020). 10.1001/jamadermatol.2020.1729 PMC8275355 32725204

[9] Reinders J (2020). Platform independent protein-based cell-of-origin subtyping of diffuse large B-cell lymphoma in formalin-fixed paraffin-embedded tissue. Sci. Rep..

[10] Altenbuchinger M (2017). Molecular signatures that can be transferred across different omics platforms. Bioinformatics.

[11] Altenbuchinger M (2017). Reference point insensitive molecular data analysis. Bioinformatics.

[12] The Cancer Genome Atlas Network. (2015). Genomic classification of cutaneous melanoma. Cell.

[13] Cirenajwis H (2015). Molecular stratification of metastatic melanoma using gene expression profiling: prediction of survival outcome and benefit from molecular targeted therapy. Oncotarget.

[14] Jayawardana K (2015). Determination of prognosis in metastatic melanoma through integration of clinico-pathologic, mutation, mRNA, microRNA, and protein information. Int. J. Cancer.

[15] Tibshirani R (1996). Regression shrinkage and selection via the Lasso. J. R. Stat. Soc. Ser. B Stat. Methodol..

[16] Kaplan EL, Meier P (1958). Nonparametric estimation from incomplete observations. J. Am. Stat. Assoc..

[17] Johnson WE, Li C, Rabinovic A (2007). Adjusting batch effects in microarray expression data using empirical Bayes methods. Biostatistics.

[18] Bedognetti D (2013). CXCR3/CCR5 pathways in metastatic melanoma patients treated with adoptive therapy and interleukin-2. Br. J. Cancer.

[19] Harlin H (2009). Chemokine expression in melanoma metastases associated with CD8+ T-cell recruitment. Cancer Res.

[20] Barbai T, Fejős Z, Puskas LG, Tímár J, Rásó E (2015). The importance of microenvironment: the role of CCL8 in metastasis formation of melanoma. Oncotarget.

[21] Wagner M, Steinskog ES, Wiig H (2019). Blockade of lymphangiogenesis shapes tumor-promoting adipose tissue inflammation. Am. J. Pathol..

[22] Strbenac D (2019). Melanoma Explorer: a web application to allow easy reanalysis of publicly available and clinically annotated melanoma omics data sets. Melanoma Res.

[23] Waldron, L. et al. Comparative meta-analysis of prognostic gene signatures for late-stage ovarian cancer. JNCI: J. of the Natl. Cancer Inst. 106, dju049. https://academic.oup.com/jnci/article/106/5/dju049/2606979 (2014). 10.1093/jnci/dju049 PMC4580554 24700801

[24] Yoshihara K (2012). High-risk ovarian cancer based on 126-gene expression signature is uniquely characterized by downregulation of antigen presentation pathway. Clin. Cancer Res..

[25] Tothill RW (2008). Novel molecular subtypes of serous and endometrioid ovarian cancer linked to clinical outcome. Clin. Cancer Res..

[26] Peloquin JM (2016). Characterization of candidate genes in inflammatory bowel disease - associated risk loci. J. Clin. Investig. Insight.

[27] McShane LM (2013). Criteria for the use of omics-based predictors in clinical trials: explanation and elaboration. BMC Med.

[28] Qin D (2021). Predict colon cancer by pairing plasma miRNAs: establishment of a normalizer-free, cross-platform model. Front. Oncol..

[29] Eisenberg E, Levanon EY (2003). Human housekeeping genes are compact. Trends Genet.

[30] R Core Team. *R: A language and environment for statistical computing*. (2019).

[31] Nickles, D., Sandmann, T., Ziman, R. & Bourgon, R. NanoStringQCPro: Quality metrics and data processing methods for NanoString mRNA gene expression data. https://www.bioconductor.org/packages/release/bioc/html/NanoStringQCPro.html (2017).

[32] Ritchie ME (2015). limma powers differential expression analyses for RNA-sequencing and microarray studies. Nucleic Acids Res.

[33] Strbenac D, Mann GJ, Ormerod JT, Yang JYH (2015). ClassifyR: an R package for performance assessment of classification with applications to transcriptomics. Bioinformatics.

[34] Friedman J, Hastie T, Tibshirani R (2010). Regularization paths for generalized linear models via coordinate descent. J. Stat. Softw..

