# Supplementary material for: Cross-Platform Omics Prediction procedure: a statistical machine learning framework for wider implementation of precision medicine
Source: NPJ Digit Med. 2022 Jul 4;5:85. doi: 10.1038/s41746-022-00618-5 (PMC9253123; doi:10.1038/s41746-022-00618-5)
Supplement: Supplementary file 1 — Supplementary materials + Supplementary table 1 and 2 [file 41746_2022_618_MOESM1_ESM.pdf]

Supplementary Notes

Data collection description

Melanoma data

We collected five melanoma datasets mostly consisting of late-stage samples, see Supplementary Table 1. Three of these data are samples from the Melanoma Institute Australia (MIA) and the other two are publicly available data. Of the three MIA data, one is measured using Illumina microarray technology and two are measured using our customised NanoString assay as described in the 'Melanoma molecular signature assay' section.

1. **MIA-Microarray**: A published gene expression study (Illumina platform) with 45 stage III melanoma subjects from Melanoma Institute Australia.

2. **MIA-NanoString**: An in-house gene expression data constructed in 2018 using the customised NanoString assay. The 45 samples are the same as the MIA-Microarray cohort described above.

3. **MIA-Validation** This independent validation cohort contains 46 samples that are age-matched and have similar characteristics to the MIA - NanoString data used to validated the CPOP procedure. Due to manufacturer error, 12 genes are unavailable to be run in this cohort and treated as missing.

4. **TCGA**: We downloaded the RNA-Seq data consisted of 472 samples from the TCGA[1] on 28th July 2017 using the `TCGABiolinks` package[2] in R[3] and Bioconductor[4]. We processed the data into log2-FPKM values and only retained 458 samples with survival times and status recorded. Out of these samples, 169 samples are labelled as Stage III, and after removing 30 samples that MIA has contributed to, we are left with a total of 139 independent samples to be used in the evaluation of survival analysis.

5. **Sweden**: This is a published microarray study from Cirenajwis et. al.[5]. We retained all 210 samples for survival-based evaluations as there is a lack of cancer staging information in the data. Processed data was downloaded on 12 January 2020 via the `GEOquery` package[6].

Ovarian cancer data

The curation of this data collection closely follows from Waldron et. al.[7] and the analysis pipeline from Yoshihara et. al.[8]. All data are downloaded through the `cureatedOvarianCancer` Bioconductor package[9]. Out of the ten datasets in Table 2 of Waldron et. al.[7], we make some key modifications to the selection of data:

- leaving out Konstantinopoulos et. al.[10] data for its incomplete sample annotations;

- leaving out Dressman et. al.[11] data as this article was retracted;
- swapping the TCGA microarray data[12] for the RNA-Seq data. And we further subset the RNA-Seq cohort to those at tumour stage of III and IV with first metastasis recurrence; and
- adding one extra data, abbreviated as "Japan B" from Yoshihara et. al.[8].

We focus on the 126 gene signature reported in Yoshihara et. al.[8] and select genes that are present in all nine datasets. This results in 94 genes with corresponding 4,371 log-ratio features. Samples in this ovarian data collection are described in Supplementary Table 2.

Inflammatory bowel disease data

This data is measured on a NanoString platform with genes originally selected to study disease-associated risk loci in inflammatory bowel disease (IBD) by Peloquin et. al.[13]. We try to classify all 983 samples as either inflamed or not inflamed learning from 712 genes. The original authors provided the raw NanoString data on Gene Expression Omnibus repository under the accession number GSE73094. We perform log2-transformation on the raw counts data. This IBD data is chosen as the experiment extends over a few years with obvious batch effect as the chemical reagent was changed twice. This change in the use of reagent creates three batches, IBD2 ($n = 303$), IBD3 ($n = 295$) and IBD4 ($n = 385$) that mimics the implementation challenge such prognostic (or risk) models will face when it is implemented in a prospective setting. Supplementary Fig. 13 shows the batch effect in this IBD via a sample boxplot and a principal component analysis (PCA) plot. Previous efforts in addressing the stability in data quality is through normalisation techniques, for example, in Molania et. al.[14]. CPOP distinguishes itself from normalisation techniques through the use of log-ratios and making predictions simultaneously.

Cross-Platform Omics Prediction (CPOP) methodology

Cross-Platform Omics Prediction (CPOP) is a procedure that enables sample prediction across gene expression datasets with different scales (e.g. different sample means). We will use the generic phrase of "scale difference" to encompass all situations where multiple gene expression data exhibit different scales in the data due to, for example, the use of different experimental instruments/platforms or drifts in measurements in a prospective setting. We use the term 'biomarker' and 'feature' interchangeably. We will use the term *predictive* in a statistical sense and the term *predictive markers* in a generic way referring to all forms of biomarkers whether they are diagnostic, prognostic or predictive. We use the term *training set* interchangeably with *reference set* (or *sets*), and restrict usage of the term *test set* or *validation set* to situations with known patient outcome i.e. to situations where we are assessing or comparing the performance of CPOP. We use the term *test sample* or *validation*

sample when the unknown subjects are to be predicted.

A major consideration in developing CPOP is to make predictions on a single-sample without normalisation or combining it with additional data. The CPOP procedure has the following three key characteristics:

1. CPOP uses (log-)ratios of genes as biomarkers (features), which are more stable than using individual gene expression values (see Step 2 of CPOP).

2. CPOP uses Elastic Net models to perform feature selection using weights proportional to the stability of features across more than one data set. This allows the selection of common predictive markers (see Step 3 of CPOP).

3. CPOP selects for features with high similarity in their between-data estimated effects (Step 4 of CPOP).

The use of (log-)ratios is not a new idea, but its usage in the context of patient outcome prediction challenges the common practice of using only genes as features. The calculation of these (log-)ratios is intended to obtain a quantitative measure for the relative differences between original omics features (e.g. genes), which we have found to be better preserved across different patient cohorts and data generation platforms. A key assumption in using these (log-)ratios is that the omics platforms in question can unbiasedly estimate the relative expression level of features. The prefix of 'log' simply reflects the prevalence of the log-transformation in dealing with omics data in practice. It is known that $\log(A/B) = \log(A) - \log(B)$ for positive values of A and B, thus, these log-ratios are able to quantify the difference in expression levels of A and B, provided the data is available on a log scale. In practice, there will be situations when published processed data are not log-transformed but with some sensible transformation on the raw measurement values. In such a case, it is assumed that these transformations will still be able to provide a sensible quantification of the relative difference between the two features A and B (e.g. subtracting one feature's value from another). We acknowledge that the (log-)ratio transformation is not suitable to be applied on a whole genome scale directly (e.g. whole genome RNA-sequencing) due to the dimensionality associated with searching over all paired features but point out that this approach is achievable on targeted omics assays. These targeted omics assays typically provide a higher signal-to-noise ratio for candidate features that are of higher clinical relevance, and are in wide use in clinical validation, the translational work and in the implementation phase of precision medicine. We also note that this (log-)ratio construction is a surrogate for the grander concept of "relative difference between features" and is certainly not limited to just transcriptomics data (e.g. similar concept exist in protein expression).

Statistical Background

Suppose we have a gene expression data matrix $\boldsymbol{X} \in \mathbb{R}^{n \times p}$ where n is the number of samples and p is the number of genes on a gene expression platform. We define the "log-ratio matrix" as a matrix $\boldsymbol{Z}$ of dimension $\mathbb{R}^{n \times q}$ where $q = \binom{p}{2}$ and each column of $\boldsymbol{Z}$ is the pairwise

difference between two log-transformed columns in $\boldsymbol{X}$. Formally, each column of $\boldsymbol{Z}$ is given by enumerating all log-ratio features $\log(\boldsymbol{x}_l) - \log(\boldsymbol{x}_m)$ for $1 \leq l < m \leq p$. Thus, each column in the $\boldsymbol{Z}$ matrix is the **log-ratio** of the expression values of **two genes**. For the given log-ratio matrix $\boldsymbol{Z} \in \mathbb{R}^{n \times q}$, we denote each row of the matrix as $\boldsymbol{z}_i$ for patient $i = 1, \ldots, n$. Let $\boldsymbol{y} \in \mathbb{R}^n$ be a vector that measures each patient's clinical outcome (e.g. patient tumour shrinkage in millimetres or prognostic outcome).

The **weighted Elastic Net (WEN) model** is a regularised regression model that solves for a regression coefficient vector $\widehat{\boldsymbol{\beta}} \in \mathbb{R}^q$ with the optimisation equation:

$$\widehat{\boldsymbol{\beta}}(\boldsymbol{y}, \boldsymbol{Z}|\boldsymbol{w}, \alpha, \lambda) = \min_{\boldsymbol{\beta} \in \mathbb{R}^q} \sum_{i=1}^{n} (y_i - \boldsymbol{z}_i^\top \boldsymbol{\beta})^2 + \lambda \sum_{j=1}^{q} w_j \left[\frac{(1-\alpha)}{2} \beta_j^2 + \alpha |\beta_j| \right], \tag{1}$$

where $\lambda \in (0, \infty]$ and $\alpha \in [0, 1]$ are tuning parameters and $\boldsymbol{w} = (w_1, \ldots, w_q)$ is a sequence of weights placed on each of the q features. This will be explained in context of our cross-platform prediction later. The *first component* of Equation (1) is a linear loss function that measures the difference between the fitted value $\boldsymbol{z}_i^\top \widehat{\boldsymbol{\beta}}$ and the observed response value y_i for sample i. This component can be readily substituted with any appropriate non-linear loss function depending on the variable type of the response variables (e.g. in 'Performance evaluation' section, logistic and Cox models are used to deal with binary and survival responses, respectively). The *second component* of the equation is a penalty on the magnitude of the estimated regression coefficients $\widehat{\boldsymbol{\beta}}$. This component mixes a L_1-norm penalty and a L_2-norm penalty through the use of the α tuning parameter. The λ tuning parameter controls the total strength of penalisation in the overall equation.

CPOP procedure

In the Main Figure 1A, CPOP is presented as a five-step procedure, with the first step being data selection. This step is often context-related and one should select datasets with similar clinical phenotypes, e.g. independent samples at the same cancer stage. We describe the rest of the CPOP procedure below:

Step 1. **Data selection**: the first step of data selection is dependent on the research questions to be addressed and one should select data with similar and appropriate clinical outcomes of interest. For example, the selected cohort can consist of independent samples at the same cancer stage. In the rest of the procedure, we assume we have two gene expression data and the CPOP model training will aim to find features consistently predictive in both data.

Step 2. **Log-ratio matrices construction**: Suppose we have two gene expression data and the associated log-ratio matrices as $\boldsymbol{Z}_1 \in \mathbb{R}^{n_1 \times q}$ and $\boldsymbol{Z}_2 \in \mathbb{R}^{n_2 \times q}$, where n_1 and n_2 are the samples sizes for the two datasets. We do not impose the restriction of paired

samples across the two data, however, we assume the two data measure the same q log-ratio features or we restrict our modelling to the common q log-ratio features between two datasets. For both data, we also have a clinical outcome measurement, denoted as $\boldsymbol{y}_1 \in \mathbb{R}^{n_1}$ and $\boldsymbol{y}_2 \in \mathbb{R}^{n_2}$ associated with data 1 and 2 respectively.

Step 3. **Selecting common predictive features**: compute a sequence of non-negative weights $w_j, j = 1, \ldots, q$ that measure the column-wise statistical concordance between $\boldsymbol{Z}_1$ and $\boldsymbol{Z}_2$. Fit a WEN model for both $(\boldsymbol{Z}_1, \boldsymbol{y}_1)$ and $(\boldsymbol{Z}_2, \boldsymbol{y}_2)$ using $w_j, j = 1, \ldots, q$ to obtain estimated regression coefficients $\widehat{\boldsymbol{\beta}}_1^{(1)}, \widehat{\boldsymbol{\beta}}_2^{(1)} \in \mathbb{R}^q$ with a penalty parameter $\alpha \in (0, 1]$. Note the superscript denotes these regression coefficients are in the first step of CPOP.

Since WEN generates sparse estimates for $\alpha \neq 0$, thus it also naturally selects features from our data as those features with non-zero estimates in $\widehat{\boldsymbol{\beta}}_1^{(1)}$ and $\widehat{\boldsymbol{\beta}}_2^{(1)}$. Define a feature set $\mathcal{S}^{(1)} = \{j | \widehat{\boldsymbol{\beta}}_{1,j}^{(1)} \neq 0, \widehat{\boldsymbol{\beta}}_{2,j}^{(1)} \neq 0\}$ that collects all non-zero features selected into both models in both data.

In this paper, we primarily focus on the use of mean-difference weights: $w_j = |\operatorname{mean}(\boldsymbol{Z}_{1j}) - \operatorname{mean}(\boldsymbol{Z}_{2j})|$ for each $j = 1, \ldots, q$, whereas other choices are also available in our `CPOP` package.

Step 4. **Selecting features with between-data stability**: define $\boldsymbol{Z}_{1,\mathcal{S}^{(1)}}$ and $\boldsymbol{Z}_{2,\mathcal{S}^{(1)}}$ as the matrices that we obtain when subsetting $\boldsymbol{Z}_1$ and $\boldsymbol{Z}_2$ to only the features present in $\mathcal{S}^{(1)}$. Then, fit an unweighted ridge regression model (i.e. a WEN model with no weights and $\alpha = 0$) onto $(\boldsymbol{Z}_{1,\mathcal{S}^{(1)}}, \boldsymbol{y}_1)$ and $(\boldsymbol{Z}_{2,\mathcal{S}^{(1)}}, \boldsymbol{y}_2)$ to obtain $\widehat{\boldsymbol{\beta}}_1^{(2)}$ and $\widehat{\boldsymbol{\beta}}_2^{(2)}$. Define another feature set $\mathcal{S}^{(2)} = \{j | \operatorname{sign}(\widehat{\boldsymbol{\beta}}_{1,j}^{(2)}) = \operatorname{sign}(\widehat{\boldsymbol{\beta}}_{2,j}^{(2)})\}$.

We also include an additional step by iteratively fitting ridge regression to both $\boldsymbol{Z}_{1,\mathcal{S}^{(2)}}$ and $\boldsymbol{Z}_{2,\mathcal{S}^{(2)}}$ and in each iteration, we update the feature set $\mathcal{S}^{(2)}$ by removing all features that do not satisfy $\operatorname{sign}(\widehat{\boldsymbol{\beta}}_{1,j}^{(2)}) = \operatorname{sign}(\widehat{\boldsymbol{\beta}}_{2,j}^{(2)})$. This removal of features using the ridge models means that the size of $\mathcal{S}^{(2)}$ is non-increasing with each iteration. This iterative step terminates when there is no further reduction in the size of $\mathcal{S}^{(2)}$.

Step 5. **Final model estimation**: the final CPOP models are the unweighted ridge regression models fitted onto $(\boldsymbol{Z}_{1,\mathcal{S}^{(2)}}, \boldsymbol{y}_1)$ and $(\boldsymbol{Z}_{2,\mathcal{S}^{(2)}}, \boldsymbol{y}_2)$. We will refer to these models as $\widehat{\boldsymbol{\beta}}_1^{\text{CPOP}}$ and $\widehat{\boldsymbol{\beta}}_2^{\text{CPOP}}$, respectively. Predictions on new samples could be made by using the coefficients $\widehat{\boldsymbol{\beta}}_1^{\text{CPOP}}$ or $\widehat{\boldsymbol{\beta}}_2^{\text{CPOP}}$ or taking the average of the two to produce a singular $\widehat{\boldsymbol{\beta}}^{\text{CPOP}}$.

In some situations, $\mathcal{S}^{(1)}$ in step 3 of CPOP might not select enough predictive features as some versions of the Elastic Net models have a tendency to only select one of many correlated features and ignoring the rest[15]. The most notable example of this is the Lasso[16]. To overcome this, we can enlarge this feature set by introducing an iterative component. This

can be done by first calculating $\mathcal{S}^{(1)}$ as described above and then removing these features from the log-ratio matrices to obtain $\boldsymbol{Z}_{1,\mathcal{S}^{(1)c}}$ and $\boldsymbol{Z}_{2,\mathcal{S}^{(1)c}}$, where $\mathcal{S}^{(1)c}$ is the set complement of $\mathcal{S}^{(1)}$. We can then fit WEN onto $(\boldsymbol{Z}_{1,\mathcal{S}^{(1)c}}, \boldsymbol{y}_1)$ and $(\boldsymbol{Z}_{2,\mathcal{S}^{(1)c}}, \boldsymbol{y}_2)$ and update the feature set by adding the selected features in each iteration into $\mathcal{S}^{(1)}$. The removal of selected features in future iterations means informative correlated features are more likely to enter the feature set. The size of $\mathcal{S}^{(1)}$ is non-decreasing and empirically we find that 20 iterations are usually enough for the size of $\mathcal{S}^{(1)}$ to stabilise.

Practical implementation

Imputation: One particular important issue is the handling of missing values. CPOP model training assumes two complete data. However, if certain genes cannot be measured in a test/validation data, then imputation on the gene-level data ($\boldsymbol{X}_{\text{test}}$) will be necessary prior to the calculation of the log-ratio matrix $\boldsymbol{Z}_{\text{test}}$. We make no particular recommendation on imputation methods as there are many good methods in the statistical literature and preference can vary among practitioners. However, we highlight a special case of missingness in gene expression data, which is when a gene is not measured at all. This situation arises typically when a gene fails quality control and any numerical values are deemed as invalid. In this case, we propose to use the non-missing gene values in the two gene-level training data of CPOP ($\boldsymbol{X}_1$ and $\boldsymbol{X}_2$) to impute on $\boldsymbol{X}_{\text{test}}$. While a variety of methods can be used, in the `CPOP` package we provide a function (`impute_cpop`) that utilises the Lasso estimator from the `glmnet` package[16;17] to make this imputation. Supplementary Figure 14 extends the results in Main Figure 3a and assumed some of the genes in the TCGA data (used as validation) are missing at random. Under 100 simulations for each level of missingness, prediction values from the imputed TCGA data is compared to the prediction values from the complete TCGA data. According to this assessment, the imputation method is able to handle up to 10% of missing genes without significantly impacting on the correlation and concordance of prediction.

Setting a CPOP prediction cut-off: Applying the CPOP model to any new sample of interest will produce a predicted value similar to that of a linear regression model. In order to produce a binary class prediction (e.g. good vs poor prognosis), a single cut-off independent of between-data scale differences is needed. Here, we will set a cut-off at 0 for the linear predicted response variable $y_{\text{test},i} = \boldsymbol{z}_{\text{test},i}\widehat{\boldsymbol{\beta}}^{\text{CPOP}}$ to produce binary class predictions. This cut-off is chosen for its ease of interpretation in the context of two common types of clinical variable modelling. In binary classification, a cut-off for this linear predicted response implies a cut-off at 0.5 for the predicted probability, see section on 'Performance evaluation'. This is a threshold which a sample is equally likely to be assigned to one of two binary classes. Similarly, in Cox regression model in survival analysis, this cut-off at 0 corresponds to a cut-off at 1 for the predicted hazard ratio, see section on 'Performance evaluation'. Here, a value great than 1 implies an at-risk sample. Though it should be noted, as with all statistical models, this cut-off is only sensible if the validation data is biologically and clinically similar to training data.

This notion of a data-independent cut-off is closely related to the challenge of between-data scale differences and forms a critical part in our evaluation. A data-adaptive choice of this cut-off on the linear prediction values, for example, using the median, could easily bias the result in assuming there is about half of high-risk incoming samples, a poor assumption in prospective testing. On the other hand, the popular area under the receiver operating characteristic curve (AUC-ROC[18]) metric for binary classification and the survival concordance index (C-index[19]) are not appropriate measures as they avoid making a cut-off and thus mask the scale differences in the data. Nonetheless, we choose to report on both of these metrics in this manuscript so comparisons may be made with other publications.

Performance evaluation

We propose an evaluation framework with an emphasis on producing between-data predictions that are robust to scale differences. Supplementary Fig. 10 summarises the evaluation framework we have. For the evaluations below, we choose to use the prediction values averaged between the two ridge models at Step 4 of the CPOP procedure. Here, we choose to focus on two most common response data seen in clinical studies:

- Binary classification response variable (e.g. good vs poor prognosis) can be modelled using the (penalised) logistic regression loss function. In logistic regression, the probability for assigning a sample i can be written as $p_{\text{test},i} = 1/[1 + \exp(-y_{\text{test},i})]$.
- Survival time response variable (e.g. recurrence-free survival) can be modelled using (penalised) Cox proportional hazard loss function. In Cox proportional hazard model, the hazard ratio can be written as $\frac{h_i(t)}{h_0(t)} = \exp(\boldsymbol{Z}_{\text{test},i}\widehat{\boldsymbol{\beta}}^{\text{CPOP}}) = \exp(y_{\text{test},i})$.

Both can be modeled using the `glmnet` package[17] (version 3.0-2).

Evaluation metrics and settings

We consider three broad classes of performance metrics to capture survival performance, classification performance and concordance performance. Supplementary Fig. 10 summarises the various metrics we use in connection with the CPOP training and testing data. In our evaluation setting, the majority of the metrics can be calculated under 100 repeated 5-fold cross-validations.

Survival performance metrics. Where survival time is available in the test data:

1. **C-index:** we take the predicted values from either a CPOP or a Lasso model and fit a classical Cox regression model (i.e. without penalisation) together with age and gender. The C-index[19] of this Cox model is reported. The C-index is defined as:

$$c = \mathbb{P}(\eta_i > \eta_j | t_i > t_j) \tag{2}$$

where t_i and t_j are survival times of sample i and j respectively and η_i and η_j are linear predicted values in the classical Cox regression model. Survival analysis is performed using the `survival` package[20].

2. **KM-plot:** we create a binary split of predicted samples at the hazard ratio of 1. This binary split means we can construct a Kaplan-Meier[21] survival plot (KM-plot) using the `survminer` package[22]. The log-rank statistic associated with this KM-plot is also reported.

3. **Log-rank test p-value:** similar to the KM-plot evaluation above, we also compute the log-rank test p-value of the binary split of predicted sample classes.

Concordance performance metrics. We propose two additional statistics to measure between-data concordance. For a validation data, we may calculate

- a re-substituted value, $\boldsymbol{Z}_{\text{test}}\widehat{\boldsymbol{\beta}}_{\text{test}}$ and use this value as the "gold-standard" against
- a prediction value $\boldsymbol{Z}_{\text{test}}\widehat{\boldsymbol{\beta}}$.

In the application to melanoma data collection, we evaluate CPOP against the competing Lasso model, where we use a Lasso-Cox model (results shown in Main Fig. 2E) for feature selection and then these features are then fitted using a ridge-Cox regression model. That is, we choose $\widehat{\boldsymbol{\beta}}$ to be the ridge regression coefficients , $\widehat{\boldsymbol{\beta}}^R$, fitted using only features selected CPOP or the Lasso. Denoting $\boldsymbol{a} = \boldsymbol{Z}_{\text{test}}\widehat{\boldsymbol{\beta}}_{\text{test}}$ and $\boldsymbol{b} = \boldsymbol{Z}_{\text{test}}\widehat{\boldsymbol{\beta}}^R$. This procedure ensures that we can make fair comparisons across two distinct feature selection methodologies. Thus, three statistics we use are:

1. **Pearson's correlation coefficient** between $\boldsymbol{a}$ and $\boldsymbol{b}$, which measures the concordance between the between-data prediction and the within-data re-substitution value. A higher positive value implies a higher quality of between-data prediction. To visualise this evaluation, the CPOP models and the Lasso-Cox models are placed on the x-axes of Main Fig. 2E and the re-substituted prediction values are placed on the y-axes and the Pearson's correlation is calculated.

2. **Identity distance** between $\boldsymbol{a}$ and $\boldsymbol{b}$, which is defined as:

$$\frac{1}{\sqrt{2}}\text{median}(|a_1 - b_1|, \ldots, |a_n - b_n|). \tag{3}$$

   This metric is based on the simple geometric fact that for a given point $(a, b) \in \mathbb{R}^2$, the quantity $\frac{1}{\sqrt{2}}(|a - b|)$ measures the perpendicular distance between the point to the identity line $y = x$. Hence, this identity distance can measure the average deviation between the within-data resubstitution values ($\boldsymbol{a}$) and the between-data prediction values ($\boldsymbol{b}$). A lower value implies a better agreement between the two quantities.

3. **Concordance correlation coefficient** between $\boldsymbol{a}$ and $\boldsymbol{b}$, which is defined as:

$$\frac{2\rho\sigma_{\boldsymbol{a}}\sigma_{\boldsymbol{b}}}{\sigma_{\boldsymbol{a}}^2 + \sigma_{\boldsymbol{b}}^2 + (\mu_{\boldsymbol{a}} - \mu_{\boldsymbol{b}})^2}. \tag{4}$$

This metric is defined by Lin[23] and it is a standard metric for evaluating reproducibility between two vectors measuring the same quantity. Its value can be expressed colloquially as:

$$1 - \frac{\text{Expected squared perpendicular deviation from the identity line}}{\substack{\text{Expected squared perpendicular deviation from the identity line,} \\ \text{assuming independence between } \boldsymbol{a} \text{ and } \boldsymbol{b}}}.$$

We will refer to this metric as "concordance" to avoid confusion with the more popular Pearson's correlation coefficient which considers the least square regression line.

Classification performance metrics. For cases where we are interested in binary classification (e.g. good vs poor prognosis) and all metrics below can be repeated applied under the 100 repeated 5-fold cross validation setting.

1. **AUC-ROC:** we use the `yardstick` package[24] to compute the AUC-ROC. Though it should be noted that this is not the most appropriate metric, its construction masks the effect of data scale differences.

2. We create a binary split of predicted samples at the predicted probability of 0.5 (e.g. predicted good prognosis class vs predicted poor prognosis class). The predicted class and true class labels defined for each data makes up a confusion matrix with four categories: true positives (TP), true negatives (TN), false positives (FP) and false negatives (FN). The predicted binary class are evaluated using classical classification statistics and computed using the `yardstick` package[24]:

   (a) **Precision** of prediction, defined as:

   $$\frac{\text{TP}}{\text{TP} + \text{FP}}. \tag{5}$$

   (b) **Recall** of prediction, defined as:

   $$\frac{\text{TP}}{\text{TP} + \text{FN}}. \tag{6}$$

   (c) **Balanced accuracy**, defined as:

   $$\frac{\text{Precision} + \text{Recall}}{2}. \tag{7}$$

(d) **The Matthews correlation coefficient** (MCC), defined as:

$$\text{MCC} = \frac{\text{TP} \times \text{TN} - \text{FP} \times \text{FN}}{\sqrt{(\text{TP} + \text{FP})(\text{TP} + \text{FN})(\text{TN} + \text{FP})(\text{TN} + \text{FN})}}. \quad (8)$$

(e) F_1 **statistic**, defined as:

$$F_1 = \frac{2\text{TP}}{2\text{TP} + \text{FP} + \text{FN}}. \quad (9)$$

All of these metrics range from 0 to 1. In the application to melanoma data collection (results shown in Main Fig. 3B), we use the CPOP model to make prediction on the MIA-validation 46 samples (reduced from five batches each consist of 12 samples by removing technical replicates).

Evaluation on sex-imbalance in melanoma data

In melanoma, there is a sex-imbalance with males typically associated with more severe outcomes[25;26]. Given that the melanoma patients we considered in our study are mostly in Stage III, it is not surprising that we have observed more males in our study as shown in Table 1. In light of this imbalance due to biology, we evaluated the fairness of prediction results by calculating the equalised odds for the two sexes, males and females. Predicted outcomes are considered as fair if the sensitivities in the subgroups are close to each other (i.e. the equalised odds is close to 1). The group-specific sensitivities indicate the number of the true positives divided by the total number of positives in that group. This calculation is implemented in the `fairness` package[27]. For the CPOP model presented in Main Figure 3, for the TCGA dataset, the equalised odds for females and males are 1.000 and 0.957, respectively. For the Sweden dataset, the equalised odds for females and males are 1.000 and 0.987, respectively. Thus, we evaluated the CPOP algorithm to be fair between the two sexes and the prediction accuracy and outcome from our CPOP model has not been impacted by this sex imbalance.

Evaluation on ovarian data collection

We apply the CPOP procedure with a penalised Cox loss function on the Japan A[8] and Tothill[28] data as the dual training set [1]. This collection of ovarian data is heterogeneous (Supplementary Fig. 11) in terms of survival times. Through careful data curation, we selected a subset of data where the range of survival times overlap between multiple data, however, the degree of separation by survival status still varies from data to data. Thus, this heterogeneity still impacts our analytics.

[1]Due to instability in the coefficient estimates in the second step of CPOP, we use an alternative approach of retaining features with estimated coefficients within 0.5 units with each other.

Evaluation on inflammatory bowel disease data

Treating IBD2 and IBD3 (raw data) as the training set, we apply the CPOP procedure with a penalised logistic loss function on the inflammation status of the samples. The selected features are then refitted back on IBD2 and IDB3 separately using ridge regression. These ridge regression models are then used to predict on the inflammation status of samples in IBD4 (Main Fig. 4C).

Supplementary Discussion

Additional remarks on scale differences between datasets

Given two arbitrary omics data measuring the same set of features, there is a very small chance that these two data will have equal scale. Main Fig. 1B provides an illustration showing boxplots of five melanoma gene expression data with four clear differences in scale. This is because omics features are typically measured on a relative scale with unit-less numerical values that are proportional to some molecular units. Technical batch effect across omics data of different origins is a classical example of this inconsistency and its presence in data is a key reason as to why omics data of different origins cannot be readily combined for the implementation of a clinical prediction work. Failing to correct for this scale difference in the data can produce misleading interpretation in the final predicted values.

For example, in Supplementary Fig. 3, we compute a Lasso model from samples in MIA - Microarray, MIA - NanoString, TCGA Stage III samples[1] and Sweden[5] samples. Then, for the TCGA stage III samples and Sweden samples, we compute the re-substituted prediction values. These values, drawn on the y-axis, are considered as gold standard of what the estimated risk probabilities should be from independent data. The prediction values from MIA - Microarray-trained Lasso model and MIA - NanoString-trained Lasso model are then plotted on the x-axis and coloured. Clearly, depending which data is used in the training of the Lasso model, we may arrive at different scale in the prediction. This comparison is particularly illustrative of the challenge associated with prediction in the presence of gene scale difference, as Main Fig. 1C shows MIA - Microarray and MIA - NanoString share high concordance for matched samples and yet their prediction values can show a large amount of variation.

Existing statistical methods aim to resolve gene scale differences through normalisation which inevitably requires estimation of data specific scales (e.g. mean and median) across all patient samples and a way to combine the samples. This procedure is often restrictive, since in a prospective experiment, it is not clear how a single sample should be combined with an existing study cohort (e.g. samples from previous studies frozen as a reference bank). Combining a single-patient's data with different cohorts and performing normalisation could lead to different predicted values for the same patient depending on the choice of the cohort.

The most popular method for adjusting data scales between different datasets is through the use of normalisation and this type of method can be divided into two broad categories: z-score standardisation and between-data normalisation. We refer to z-score standardisation as a pre-processing step where each omics predictor is centred at 0 and its sample variance is scaled to 1. This procedure has been widely used[7;29;30;31;32;33]. On the other hand, between-data normalisation aims to correct the statistical distributions of genes in validation data to be similar to that of training data. Rudy et. al.[34] provides an evaluation of nine different between-data normalisation methods and more recent updates on this kind of normalisation include Taroni et. al.[35] and Thompson et. al.[36]. Through the act of combining multiple data and calculating summary statistics like sample-wise mean, both of these methods introduce interdependencies between the samples and may not be suitable for processing and predicting on a single omics profile in Criterion 15 of McShane et. al.[37]. Though within-sample standardisation methods such as in Le Cao et. al.[38] have the potential to bypass some of these constraints, in this manuscript, we will operate under the assumption that re-normalisation for incoming samples together with existing cohort is not practical due to constraint with consent, data security or other reasons.

Data normalisation alone is not enough to address the various challenges in implementing precision medicine utilising omics data with additional considerations relating to the model stability and reproducibility also being necessary. We will demonstrate this effect using a popular normalisation method, ComBat[39]. We first took the log-ratio matrices of MIA-Microarray, MIA-NanoString and TCGA from Supplementary Table 1 and performed the ComBat normalisation, available through the `sva` package in `R`. See Supplementary Figure 12 for the sample-wise boxplots before and after the ComBat normalisation. The normalised data is then split back to their original respective sources. Lasso models are fitted on the MIA-Microarray-normalised and MIA-NanoString-normalised data and the TCGA data is used as the validation data for both Lasso models. The prediction values from both Lasso models are then compared against each other. The CPOP model in Main Figure 3 was used again to compare the prediction values between MIA-Microarray and MIA-NanoString. Note that in the case of CPOP, no normalisation was performed on the log-ratios. Supplementary Figure 13a shows the prediction values from the Lasso models exhibit clear bias away from the identify line. This comparison of prediction values was designed to give the normalised data an advantage, as the TCGA validation data was also used in the normalisation, which created data leakage in the between-data modelling. Despite this, we see that the Lasso prediction values exhibit bias away from the identity line (red line). We compare this against Supplementary Figure 13b, where the CPOP prediction values are shown to have much lower bias. Thus, we see that the instability of prediction is not always fully addressed by normalisation.

To confirm that the observed bias effect is not due to random seeds in computation, 100 bootstrap samplings were performed on the samples of the normalised and unnormalised data with the Lasso and the CPOP model separately. The concordance and correlation between the predicted values are used to summarise the presence of bias as shown in the

boxplots in Supplementary Figure 13C and Supplementary Figure 13D, respectively.

While there are numerous modelling approaches we could have taken, for example, rank-based approach such as Eddy et. al.[40] and Afsari[41] or tree-based approach like Breiman et. al.[42] and Ishwaran et. al.[43]; we ultimately opt for a regression-based modelling approach because we want to efficiently utilise all gene expression information rather than summarising into ranks and wish to place a greater emphasis on model/feature interpretability.

Supplementary Figures

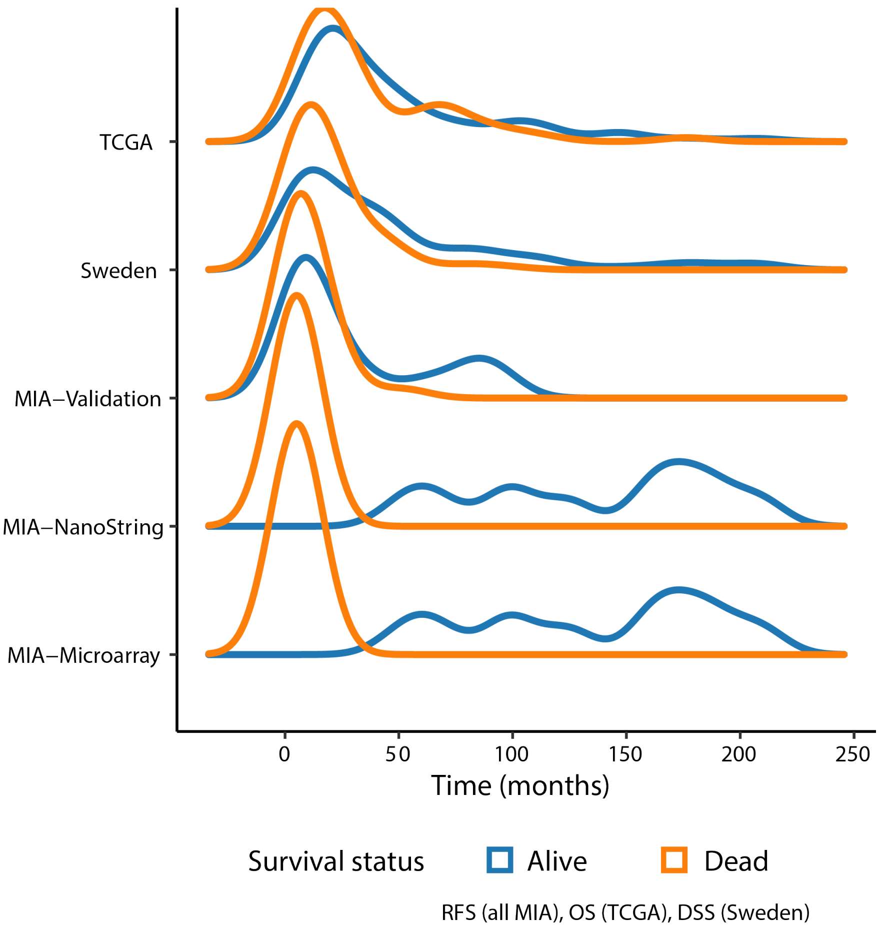

Supplementary Figure 1: Distribution of the survival time for the melanoma data collection, stratified by survival status. All data illustrated here use disease-specific survival times or recurrence-free survival except for TCGA data where overall survival is used.

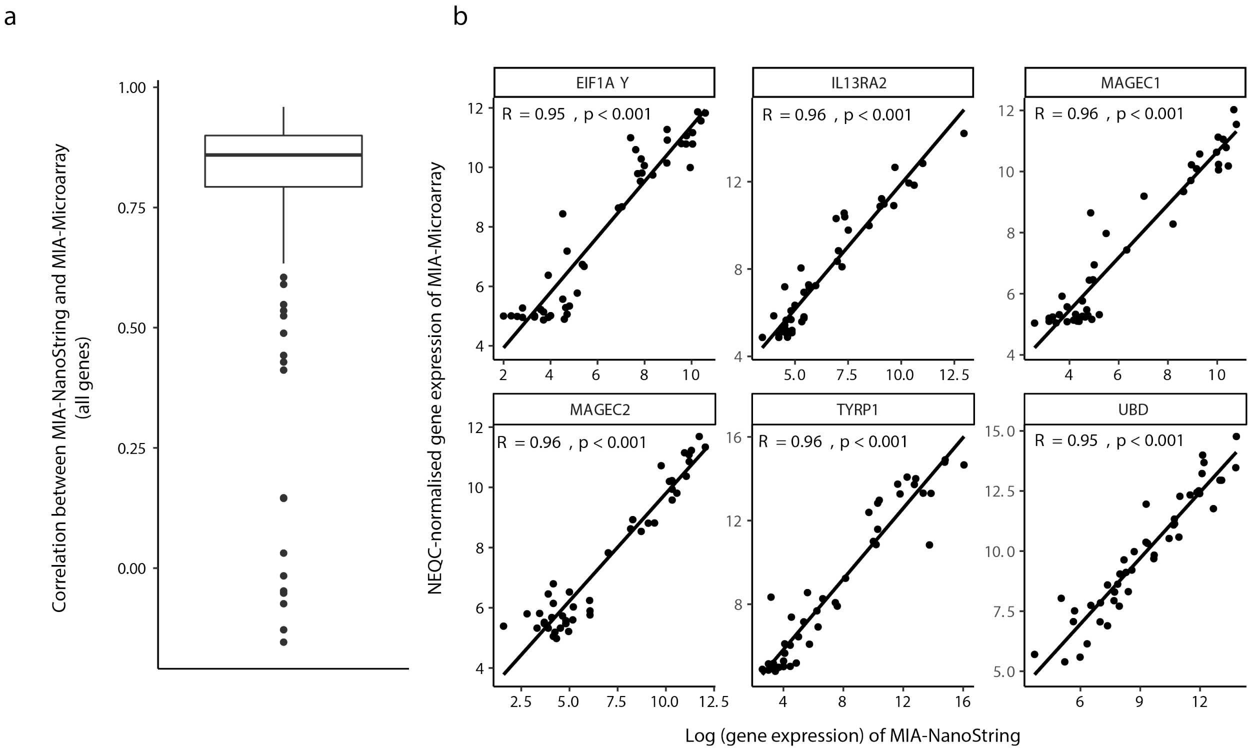

Supplementary Figure 2: **a** Boxplot of Pearson's correlation of gene expression values between MIA-Microarray and MIA-NanoString for 192 common genes. **b** Scatter-plot of six genes with the highest correlation across the two data.

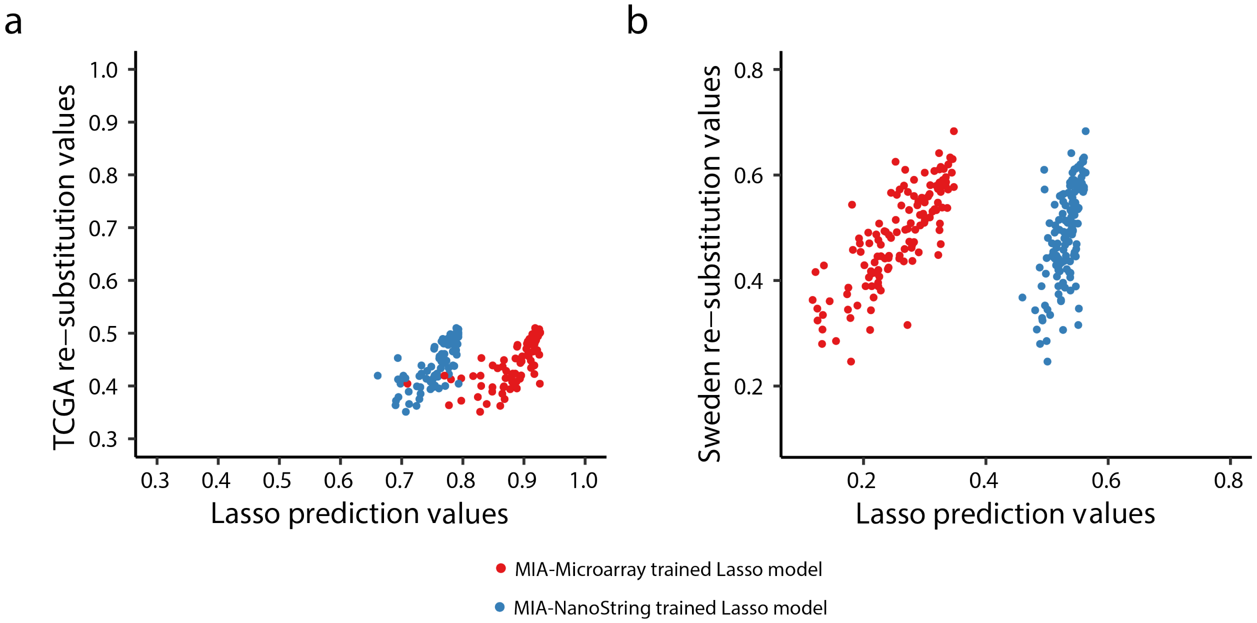

Supplementary Figure 3: These plots highlight the statistical challenges of Lasso regression, where scale difference in the data can lead to scale difference in the prediction which will affect the translational interpretation. On the x-axis, we plot the prediction values from a Lasso model trained on MIA-Microarray (red points) and another trained on MIA-NanoString (blue points), with both models making predictions on the TCGA (panel **a**) and Sweden (panel **b**) samples. On the y-axis, we plot the re-substituted Lasso models where we trained and tested on the TCGA data and Sweden. As the re-substituted values (y-axis) are identical between data platform, we can then fairly compare the scale of prediction values between the MIA-Microarray-trained model and the MIA-NanoString-trained model (x-axis) and see an obvious scale difference between the two. Furthermore, both of these between-data prediction values are on a very different centering/scale to the re-substituted ("gold-standard") values from TCGA and Sweden.

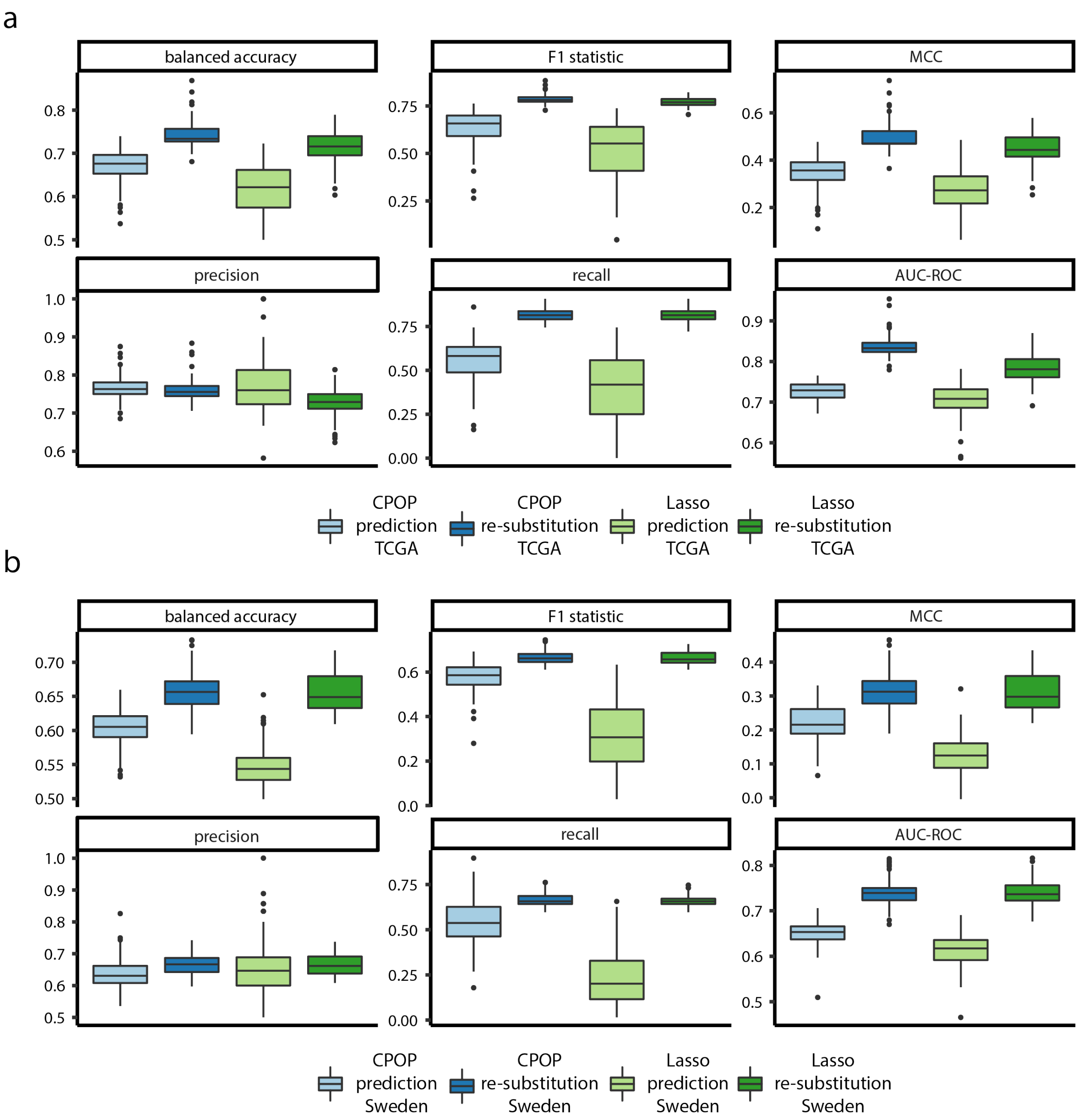

Supplementary Figure 4: We perform 20 repeated 5-fold cross-validation to select 100 different sets of features where each CPOP and Lasso feature set is constructed from 80% of MIA-Microarray and MIA-NanoString samples. We compare the CPOP and Lasso predicted probabilities performance (light blue and light green) in for TCGA and Sweden in panel **a** and **b** respectively. We also add the within-data re-substituted values for CPOP and Lasso for reference (darker blue and darker green). While within-data performance of CPOP and Lasso are similar, CPOP consistently perform better than Lasso in terms of between-data performance.

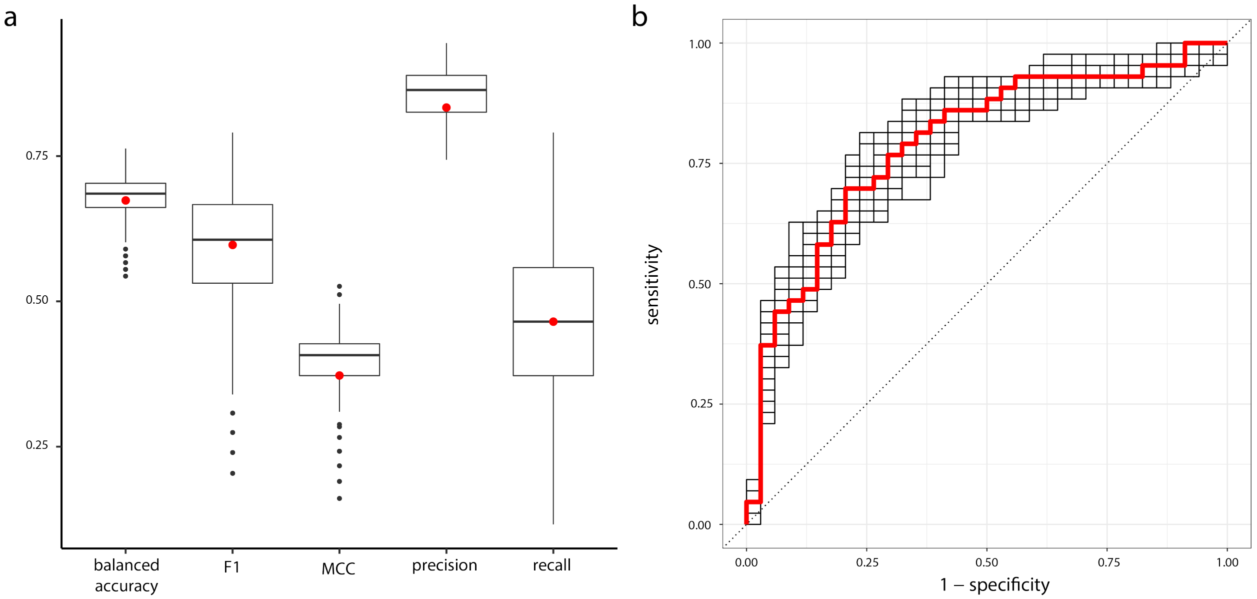

Supplementary Figure 5: Assessment of prediction model with missing values. We introduce missing values into the TCGA data by randomly removing 5 genes and use the training model to impute the missing genes before calculating the log-ratio matrices and corresponding CPOP prediction. The process randomly removing of 5 genes is repeated 100 times. Panel **a** illustrates the prediction performance using five difference performance metrics (balance accuracy, F1, MCC, precision and recall). The red points represent the prediction performance on the complete TCGA without any missing values. Panel **b** shows the ROC curves for the prediction performance on imputed data (black line) where we predict on the TCGA data with 5 genes randomly removed and the complete TCGA data (red line).

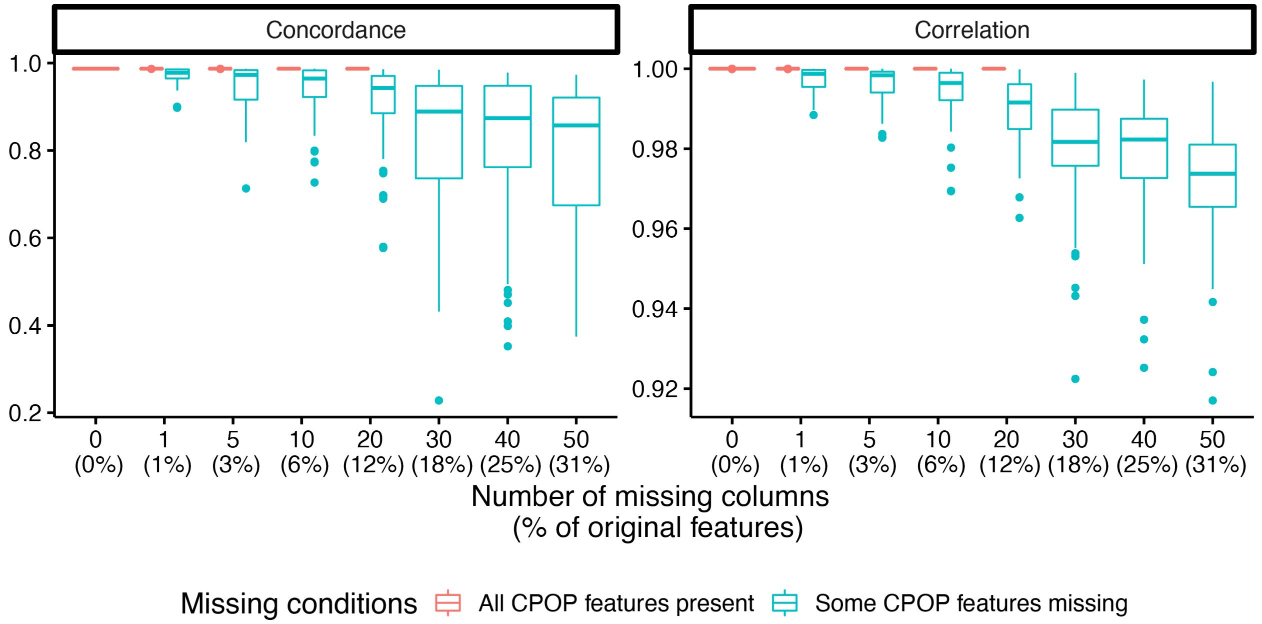

Supplementary Figure 6: Assessment of the impact of missing features in predicting TCGA data. Assuming missing features at random, the described imputation method is able to handle up to approximately 20 of the 163 (12%) of missing columns without significantly impacting on the correlation and concordance of prediction.

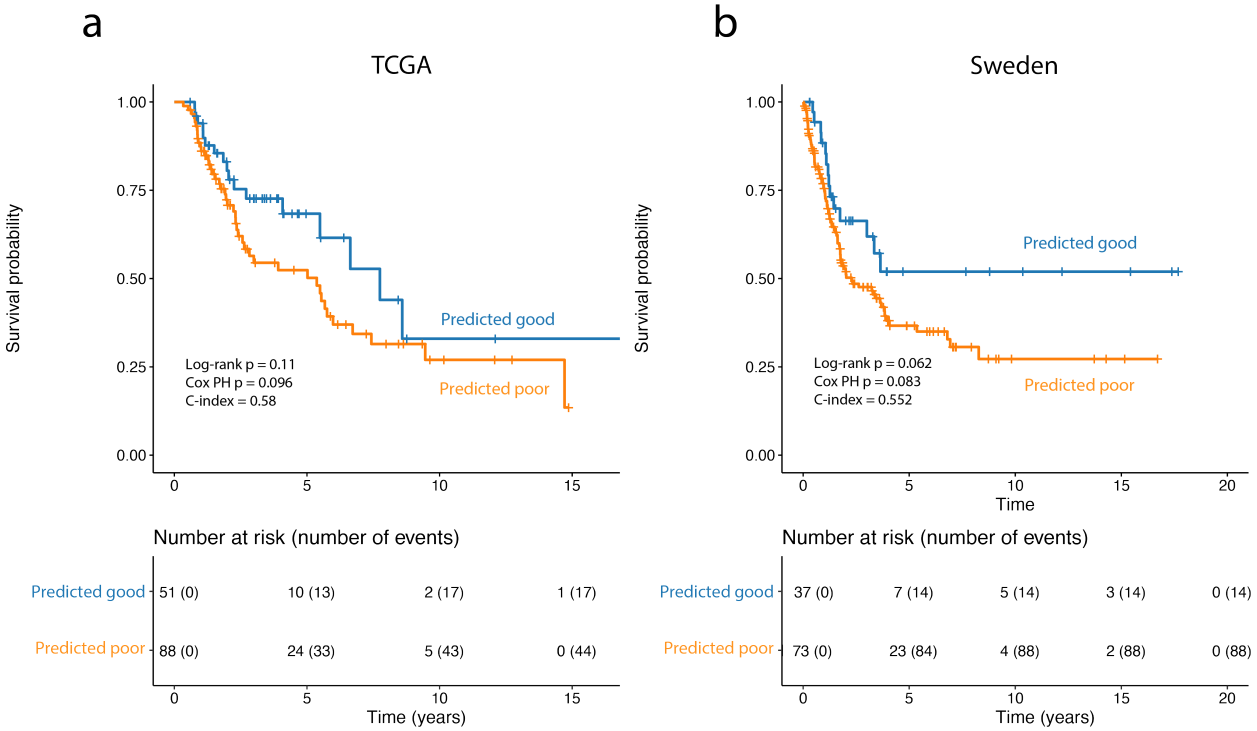

Supplementary Figure 7: Kaplan-Meier plots showing the prediction performance of the Lasso model on the TCGA (**a**) and the Sweden data (**b**), respectively. The Lasso model used both MIA-Microarray and MIA-NanoString as the training data. Compared to this Lasso model, the CPOP model in Main Figure 3 is able to achieve better separation of predicted prognosis groupings on the TCGA and Sweden validation data.

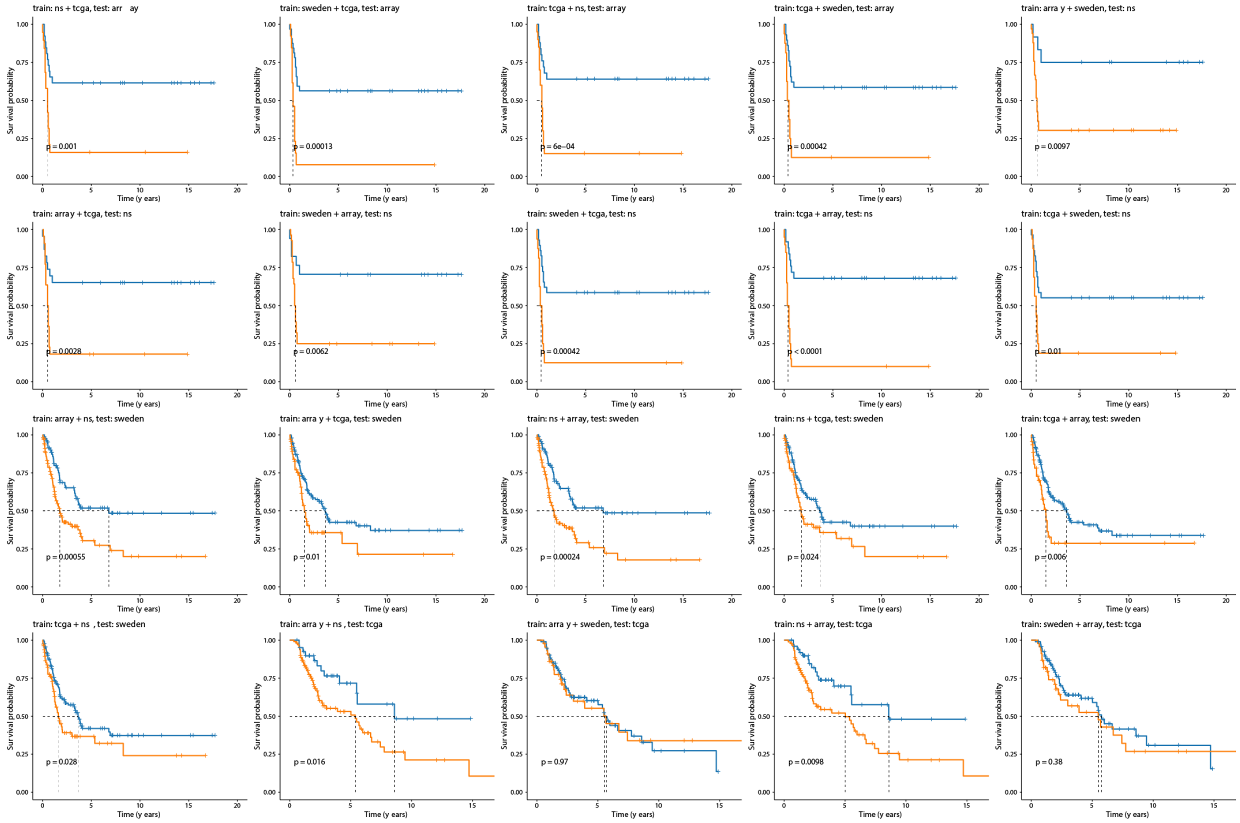

Supplementary Figure 8: Assessment of melanoma data with survival performance metrics. Kaplan-Meier plots show a significant difference between the predicted good (blue line) and poor (orange line) prognostic classes on different training-testing pairs from four of the melanoma data collection (MIA-Microarray, MIA-NanoString, TCGA and Sweden). Here, we show that the general applicability of the CPOP procedure through KM-plots of all 24 combinations of training-testing set of the MIA-Microarray, MIA-NanoString, TCGA and Sweden data. While not every training and testing combination shows a statistical difference, a majority (19 out of 24, or 79%) of pairs did.

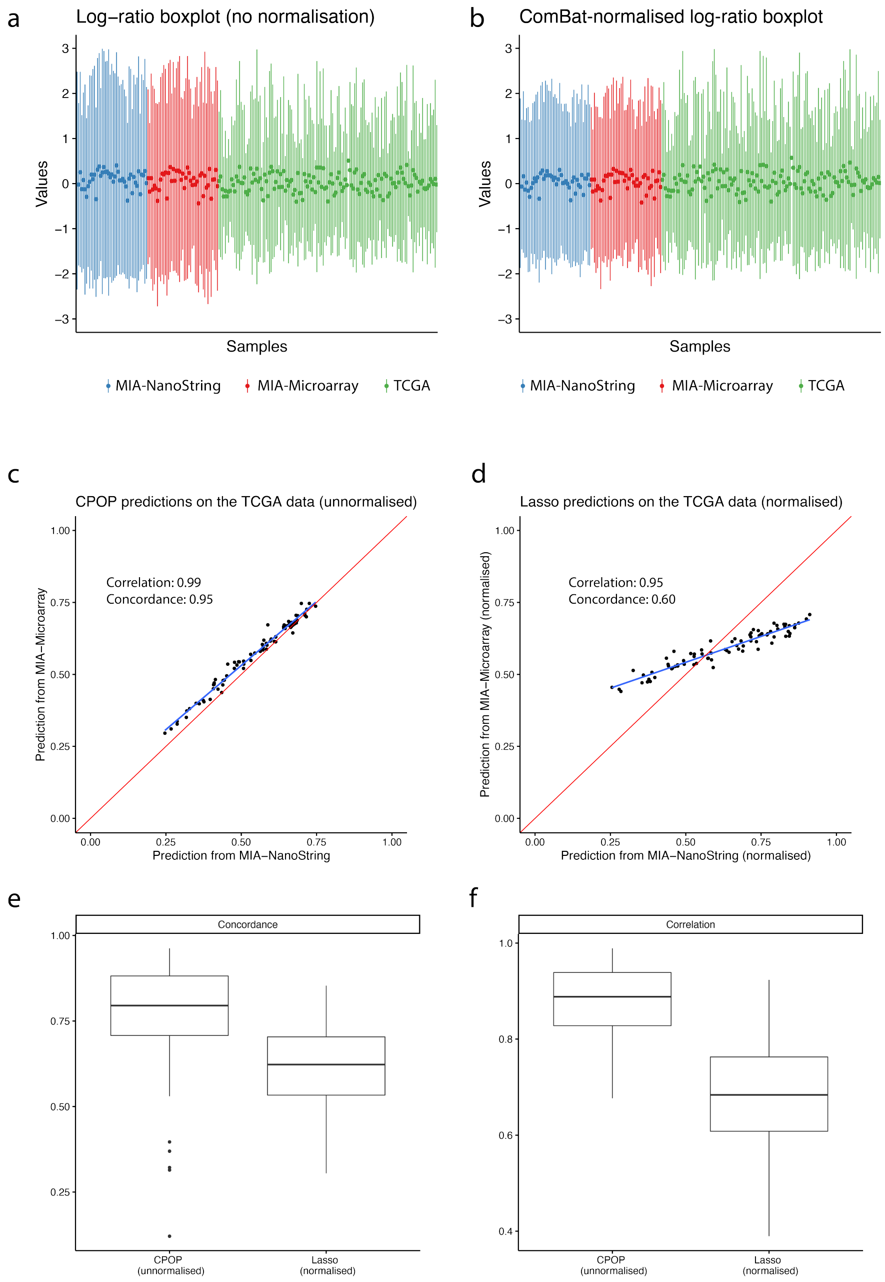

Supplementary Figure 9: **a** Quartile plot of unnormalised log-ratios across three melanoma data. **b** Quartile plot of ComBat normalised log-ratios across the same three melanoma data. **c** Comparing CPOP prediction values on the TCGA unnormalised data. Each point represents a TCGA sample. **d** Comparing Lasso prediction values on the TCGA Combat normalised data. We note the increased bias compared to panel A. **e** and **f** Under 100 bootstrap sampling on the MIA-NanoString and MIA-Microarray data (i.e. the training data), we repeatedly calculate the concordance and correlation between the prediction values for the TCGA data, respectively. The Wilcoxon rank sum test p-value for the concordance and correlation metrics are 1.2e-15 and <2e-16 respectively.

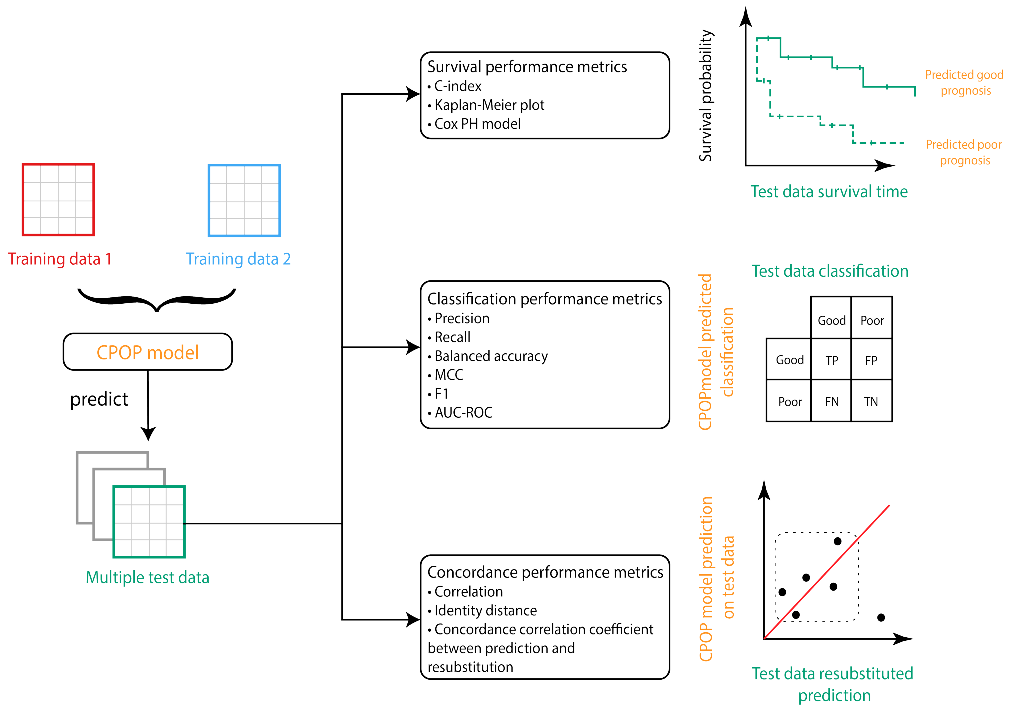

Supplementary Figure 10: Schematic illustration of the evaluation framework for CPOP using three different classes of metrics.

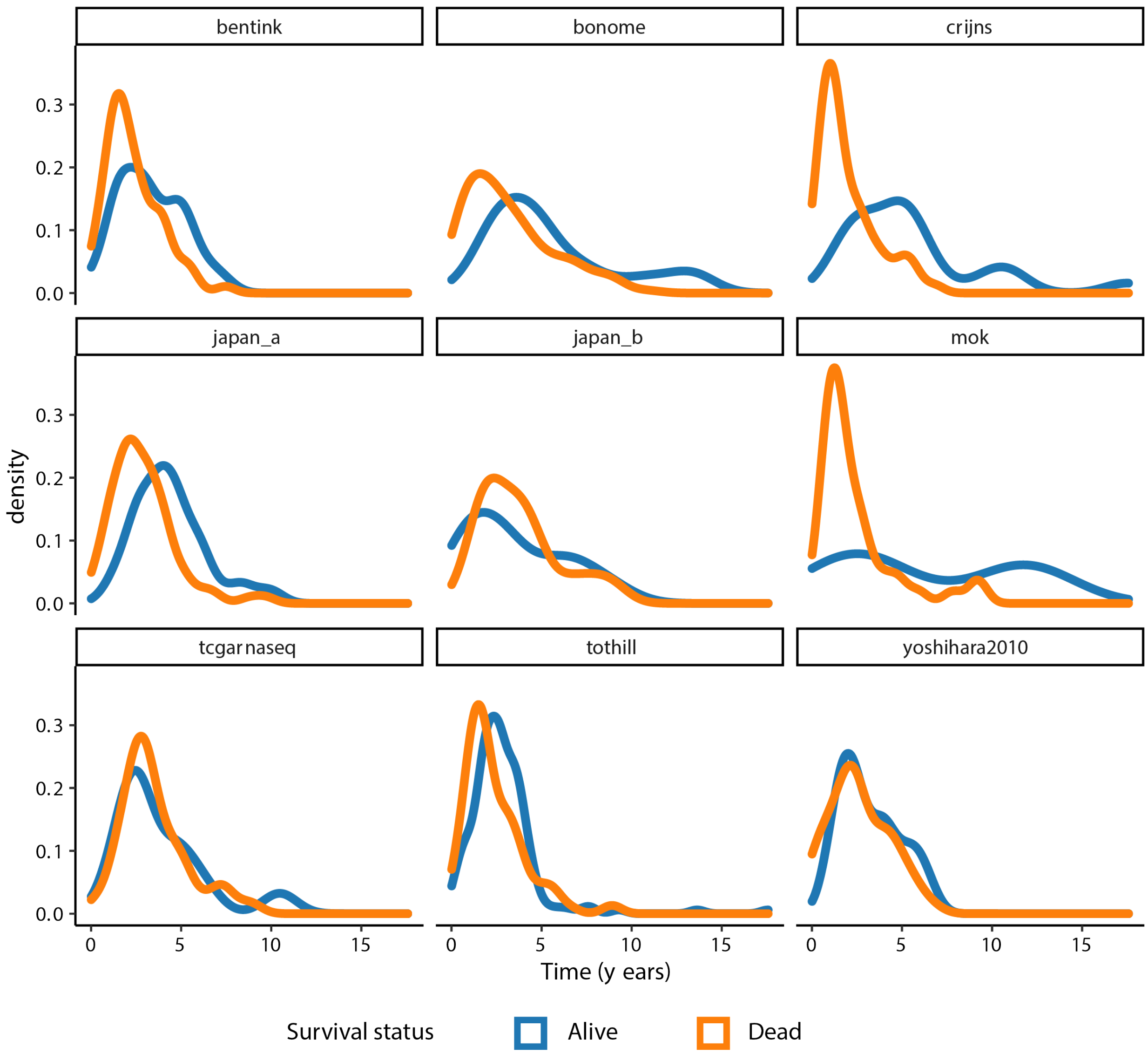

Supplementary Figure 11: Distribution of the survival time for the Ovarian cancer data collection, stratified by survival status. All data illustrated here uses overall survival time.

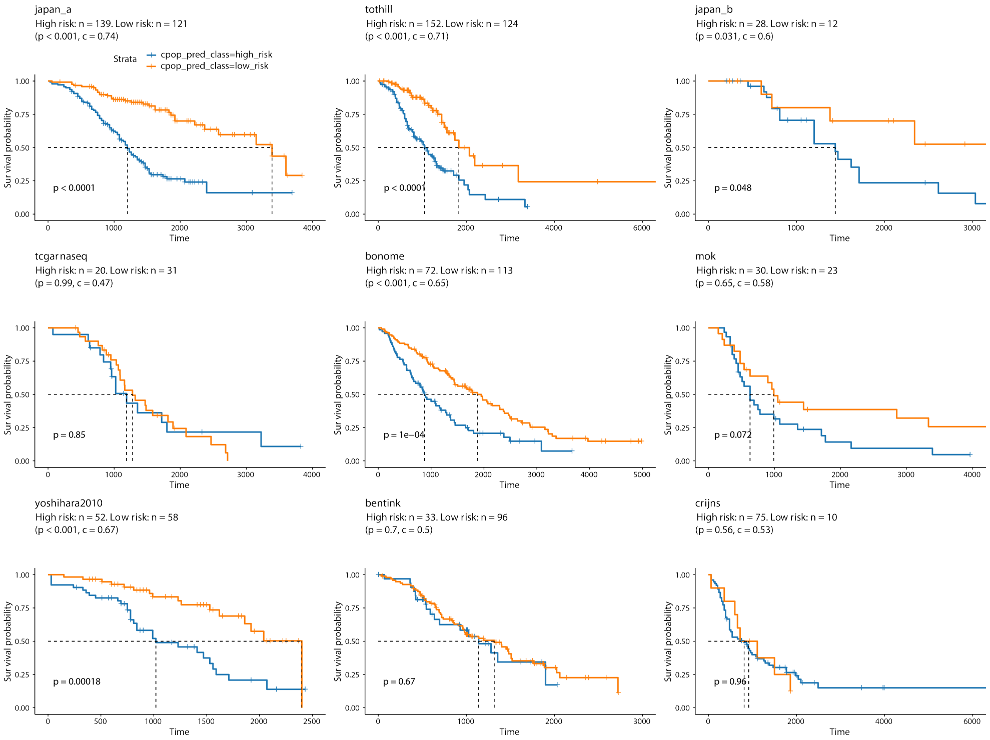

Supplementary Figure 12: Assessment of the ovarian data with survival performance metrics. Kaplan-Meier plot of CPOP prediction on all the nine ovarian data showing the survival probability between the predicted good (blue line) and poor (orange line) prognostic classes. We build the model using the CPOP procedure with a penalised Cox loss function on the Japan A[8] and Tothill[28] data. Five out of the nine data show a statistical significance between good and poor predicted outcomes. This illustrates the high predictive strength of the CPOP method. While not all data achieved statistical significance as the original publication[7], it should be noted that z-score standardisation (standardisation with mean equal to 0 and variance equial to 1) is applied on all data prior to modelling in[7]. On the other hand, our CPOP evaluation avoids this transformation for the reason that we wish to best evaluate our method in a single-sample prediction situation where z-transformation is not impossible.

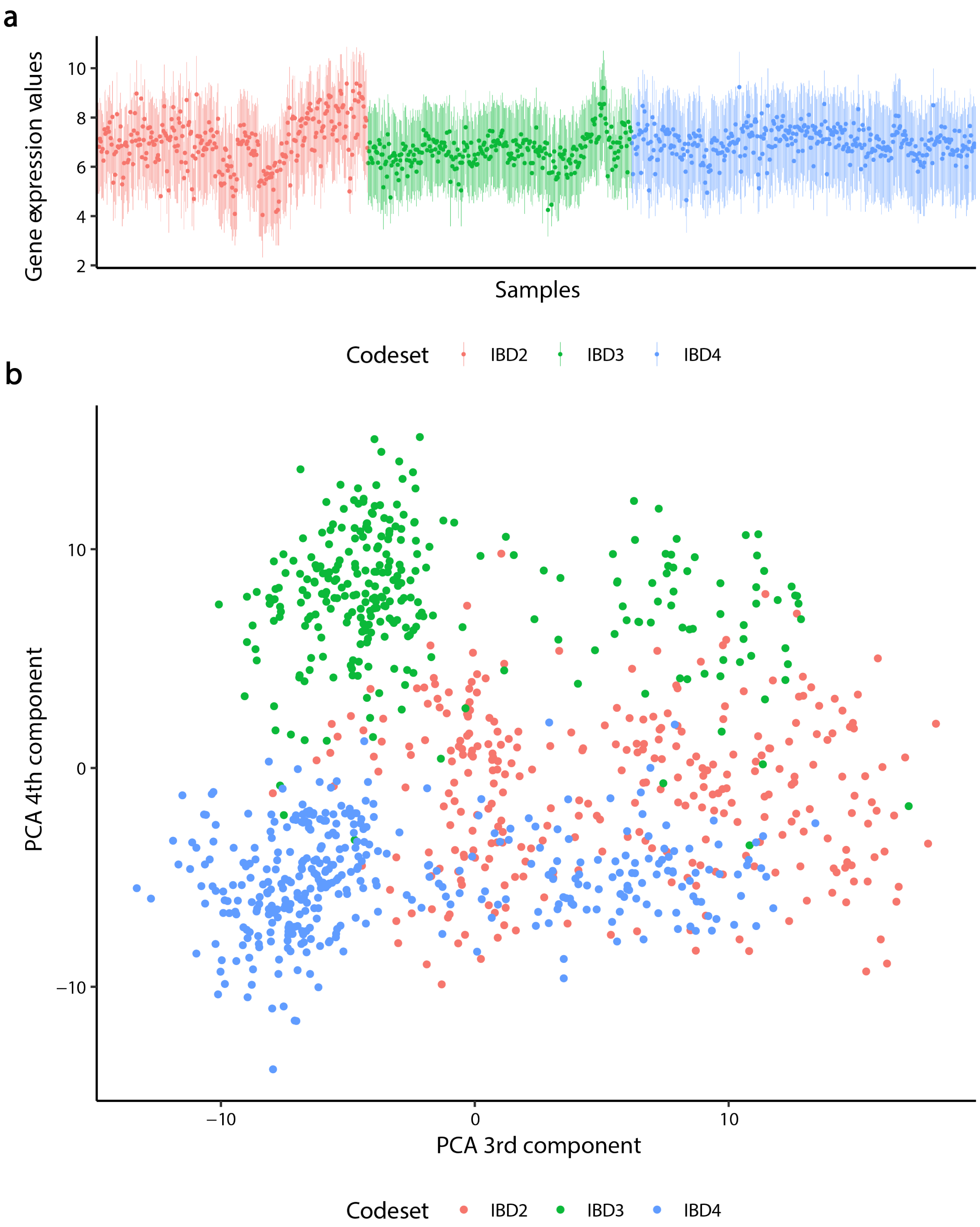

Supplementary Figure 13: **a** Quartile plot of gene expression values for the IBD data, coloured by the reagent codeset. Each sample is represented by its median (a single solid point), the 25% quantile of the gene/features (the lower end of a vertical line) and 75% quantile of the gene/features (the upper end of a vertical line). **b** PCA plot using the 3rd and 4th components, coloured by the reagent codeset.

Supplementary Tables

- Supplementary Table 1 (below): Data collection and processing summaries for five melanoma datasets, MIA-Microarray, MIA-NanoString, TCGA, Sweden and MIA-Validation. Including the data platforms, number of samples, definition of the prognostic groups, data accession number, date of download and references. OS, RFS and DSS refers to overall survival, recurrence-free survival and disease specific survival, respectively.

- Supplementary Table 2 (below): Table of samples in the ovarian data, with median survival time.

- Supplementary Table 3 (separate CSV file): Table of NanoString panel genes for stage III melanoma prognosis.

- Supplementary Table 4 (separate CSV file): Table of CPOP coefficients for stage III melanoma prognosis constructed from MIA-Microarray and MIA-NanoString.

Table 1: Data processing on five melanoma data.

	Platform	n (good)	n (poor)	Definition of prognosis groups.	Processing strategies	Accession	Date	Reference
MIA - Microarray	Illumina Human WG-6 BeadChip microarray Version 3.	19	26	Good: RFS more than 4 years and alive with no sign of relapse. Poor: RFS less than 1 year and died due to melanoma.	NEQC normalisation	GSE54467	8 Jun 2016	[44;45]
MIA - NanoString	Customised NanoString nCounter assay	19	26	Good: RFS more than 4 years and alive with no sign of relapse. Poor: RFS less than 1 year and died due to melanoma.	log2(raw counts)	GSE156030	Between 24 May 2017 and 17 Apr 2018	This paper
TCGA - SKCM	RNA-Seq	43	34	Good: survived more than the median survival time (26.9 mo). Poor: survived less than the median survival time.	log2(FPKM)	GDC-TCGA portal	21 Apr 2018	[1]
Sweden	Illumina HumanHT-12 V4.0 beadchip	67	64	Good: survived more than the median survival time (17.6 mo). Poor: survived less than the median survival time.	Normalised values from GEO	GSE65904	12 Jan 2020	[5]
MIA - validation	Customised NanoString nCounter assay	-	-	Validation cohort with 46 samples	log2(raw counts)	GSE156030	Between 5 Mar 2020 and 13 Mar 2020	This paper

Table 2: Samples and median survival time for nine ovarian data.

	Data source	Abbreviation	Median survival (months)	Number of samples
1	Yoshihara et. al.[8]	Japan A	104	260
2	Tothill et. al.[28]	Tothill	72	276
3	Yoshihara et. al.[8]	Japan B	96	40
4	Bell et. al.[12]	TCGA RnaSeq	91	51
5	Bonome et. al.[46]	Bonome	97	185
6	Mok et. al.[47]	Mok	52	53
7	Yoshihara et. al.[48]	Yoshihara2010	76	110
8	Bentink et. al.[49]	Bentink	76	129
9	Crijns et. al.[50]	Crijins	52	157

Supplementary Reference

[1] TCGA. Genomic Classification of Cutaneous Melanoma. *Cell* **161**, 1681–1696 (2015).

[2] Colaprico, A. *et al.* TCGAbiolinks: An R/Bioconductor package for integrative analysis of TCGA data. *Nucleic Acids Research* **44**, e71–e71 (2016).

[3] R Core Team. *R: A Language and Environment for Statistical Computing* (Vienna, Austria, 2019).

[4] Gentleman, R. C. *et al.* Bioconductor: Open software development for computational biology and bioinformatics. *Genome Biology* 16 (2004).

[5] Cirenajwis, H. *et al.* Molecular stratification of metastatic melanoma using gene expression profiling : Prediction of survival outcome and benefit from molecular targeted therapy. *Oncotarget* **6**, 12297–12309 (2015).

[6] Davis, S. & Meltzer, P. S. GEOquery: A bridge between the Gene Expression Omnibus (GEO) and BioConductor. *Bioinformatics* **23**, 1846–1847 (2007).

[7] Waldron, L. *et al.* Comparative Meta-analysis of Prognostic Gene Signatures for Late-Stage Ovarian Cancer. *JNCI: Journal of the National Cancer Institute* **106** (2014).

[8] Yoshihara, K. *et al.* High-Risk Ovarian Cancer Based on 126-Gene Expression Signature Is Uniquely Characterized by Downregulation of Antigen Presentation Pathway. *Clinical Cancer Research* **18**, 1374–1385 (2012).

[9] Ganzfried, B. F. *et al.* curatedOvarianData: Clinically annotated data for the ovarian cancer transcriptome. *Database* **2013** (2013).

[10] Konstantinopoulos, P. A. *et al.* Gene Expression Profile of BRCAness That Correlates With Responsiveness to Chemotherapy and With Outcome in Patients With Epithelial Ovarian Cancer. *Journal of Clinical Oncology* **28**, 3555–3561 (2010).

[11] Dressman, H. K. *et al.* An integrated genomic-based approach to individualized treatment of patients with advanced-stage ovarian cancer. *Journal of Clinical Oncology: Official Journal of the American Society of Clinical Oncology* **25**, 517–525 (2007).

[12] Bell, D. *et al.* Integrated genomic analyses of ovarian carcinoma. *Nature* **474**, 609–615 (2011).

[13] Peloquin, J. M. *et al.* Characterization of candidate genes in inflammatory bowel disease - associated risk loci. *Journal of Clinical Investigation Insight* **1**, e87899 (2016).

[14] Molania, R., Gagnon-Bartsch, J. A., Dobrovic, A. & Speed, T. P. A new normalization for Nanostring nCounter gene expression data. *Nucleic Acids Research* **47**, 6073–6083 (2019).

[15] Zou, H. & Hastie, T. Regularization and variable selection via the elastic net. *Journal of the Royal Statistical Society: Series B (Statistical Methodology)* **67**, 301–320 (2005).

[16] Tibshirani, R. Regression shrinkage and selection via the Lasso. *Journal of the Royal Statistical Society Series B* **58**, 10–14 (1996).

[17] Friedman, J., Hastie, T. & Tibshirani, R. Regularization paths for generalized linear models via coordinate descent. *Journal of Statistical Software* **33**, 1–22 (2010).

[18] Fawcett, T. An introduction to ROC analysis. *Pattern Recognition Letters* **27**, 861–874 (2006).

[19] Harrell, F. E., Lee, K. L. & Mark, D. B. Multivariable prognostic models: Issues in developing models, evaluating assumptions and adequacy, and measuring and reducing errors. *Statistics in Medicine* **15**, 361–387 (1996).

[20] Therneau, T. M. *A Package for Survival Analysis in r* (2020).

[21] Kaplan, E. L. & Meier, P. Nonparametric Estimation from Incomplete Observations. *Journal of the American Statistical Association* **53**, 457–481 (1958).

[22] Kassambara, A., Kosinski, M. & Biecek, P. *Survminer: Drawing Survival Curves Using 'Ggplot2'* (2020).

[23] Lin, L. I. A concordance correlation coefficient to evaluate reproducibility. *Biometrics* **45**, 255–268 (1989).

[24] Kuhn, M. & Vaughan, D. *Yardstick: Tidy Characterizations of Model Performance* (2020).

[25] Scoggins, C. R. *et al.* Gender-Related differences in outcome for melanoma patients. *Annals of Surgery* **243** (2006).

[26] Bellenghi, M. *et al.* Sex and gender disparities in melanoma. *Cancers* **12** (2020).

[27] Kozodoi, N. & V. Varga, T. *fairness: Algorithmic Fairness Metrics* (2021). URL `https://CRAN.R-project.org/package=fairness`. R package version 1.2.2.

[28] Tothill, R. W. *et al.* Novel Molecular Subtypes of Serous and Endometrioid Ovarian Cancer Linked to Clinical Outcome. *Clinical Cancer Research* **14**, 5198–5208 (2008).

[29] Kossenkov, A. V. *et al.* A gene expression classifier from whole blood distinguishes benign from malignant lung nodules detected by low-dose CT. *Cancer Research* **79**, 263–273 (2019).

[30] Deng, X., Xiao, Q., Liu, F. & Zheng, C. A gene expression-based risk model reveals prognosis of gastric cancer. *PeerJ* **6**, e4204 (2018).

[31] Liu, Q., Diao, R., Feng, G., Mu, X. & Li, A. Risk score based on three mRNA expression predicts the survival of bladder cancer. *Oncotarget* **8**, 61583–61591 (2017).

[32] Chen, Q. R. *et al.* An integrated cross-platform prognosis study on neuroblastoma patients. *Genomics* **92**, 195–203 (2008).

[33] Herold, T. *et al.* A 29-gene and cytogenetic score for the prediction of resistance to induction treatment in acute myeloid leukemia. *Haematologica* **103**, 456–465 (2018).

[34] Rudy, J. & Valafar, F. Empirical comparison of cross-platform normalization methods for gene expression data. *BMC Bioinformatics* **12**, 1–22 (2011).

[35] Taroni, J. N. & Greene, C. S. Cross-platform normalization enables machine learning model training on microarray and RNA-Seq data simultaneously. *bioRxiv* 118349 (2017).

[36] Thompson, J. A., Tan, J. & Greene, C. S. Cross-platform normalization of microarray and RNA-seq data for machine learning applications. *PeerJ* **4**, e1621 (2016).

[37] McShane, L. M. *et al.* Criteria for the use of omics-based predictors in clinical trials: Explanation and elaboration. *BMC Medicine* **11** (2013).

[38] Lê Cao, K. A., Rohart, F., McHugh, L., Korn, O. & Wells, C. A. YuGene: A simple approach to scale gene expression data derived from different platforms for integrated analyses. *Genomics* **103**, 239–251 (2014).

[39] Leek, J. T. *et al. sva: Surrogate Variable Analysis* (2021). R package version 3.42.0.

[40] Eddy, J. A., Sung, J., Geman, D. & Price, N. D. Relative Expression Analysis for Molecular Cancer Diagnosis and Prognosis. *Technology in Cancer Research & Treatment* **9**, 149–159 (2010).

[41] Afsari, B., Fertig, E. J., Geman, D. & Marchionni, L. switchBox: An R package for k–Top Scoring Pairs classifier development. *Bioinformatics* **31**, 273–274 (2015).

[42] Breiman, L. Random Forests. *Machine Learning* **45**, 5–32 (2001).

[43] Ishwaran, H., Kogalur, U. B., Blackstone, E. H. & Lauer, M. S. Random survival forests. *The Annals of Applied Statistics* **2**, 841–860 (2008).

[44] Mann, G. J. *et al.* BRAF Mutation, NRAS Mutation, and the Absence of an Immune-Related Expressed Gene Profile Predict Poor Outcome in Patients with Stage III Melanoma. *Journal of Investigative Dermatology* **133**, 509–517 (2013).

[45] Jayawardana, K. *et al.* Determination of prognosis in metastatic melanoma through integration of clinico-pathologic, mutation, mRNA, microRNA, and protein information. *International Journal of Cancer* **136**, 863–874 (2015).

[46] Bonome, T. *et al.* A Gene Signature Predicting for Survival in Suboptimally Debulked Patients with Ovarian Cancer. *Cancer Research* **68**, 5478–5486 (2008).

[47] Mok, S. C. *et al.* A gene signature predictive for outcome in advanced ovarian cancer identifies a survival factor: Microfibril-associated glycoprotein 2. *Cancer Cell* **16**, 521–532 (2009).

[48] Yoshihara, K. *et al.* Gene Expression Profile for Predicting Survival in Advanced-Stage Serous Ovarian Cancer Across Two Independent Datasets. *PLOS ONE* **5**, e9615 (2010).

[49] Bentink, S. *et al.* Angiogenic mRNA and microRNA Gene Expression Signature Predicts a Novel Subtype of Serous Ovarian Cancer. *PLOS ONE* **7**, e30269 (2012).

[50] Crijns, A. P. G. *et al.* Survival-Related Profile, Pathways, and Transcription Factors in Ovarian Cancer. *PLOS Medicine* **6**, e1000024 (2009).
