# Supplementary material for: Cross-Platform Omics Prediction procedure: a statistical machine learning framework for wider implementation of precision medicine
Source: NPJ Digit Med. 2022 Jul 4;5:85. doi: 10.1038/s41746-022-00618-5 (PMC9253123; doi:10.1038/s41746-022-00618-5)
Supplement: Supplementary file 4 — Reporting Summary [file 41746_2022_618_MOESM4_ESM.pdf]

nature portfolio

Corresponding author(s): Jean Yang

Last updated by author(s): Feb 26, 2022

Reporting Summary

Nature Portfolio wishes to improve the reproducibility of the work that we publish. This form provides structure for consistency and transparency in reporting. For further information on Nature Portfolio policies, see our Editorial Policies and the Editorial Policy Checklist.

Statistics

For all statistical analyses, confirm that the following items are present in the figure legend, table legend, main text, or Methods section.

n/a	Confirmed	
☐	☒	The exact sample size (*n*) for each experimental group/condition, given as a discrete number and unit of measurement
☐	☒	A statement on whether measurements were taken from distinct samples or whether the same sample was measured repeatedly
☐	☒	The statistical test(s) used AND whether they are one- or two-sided<br>*Only common tests should be described solely by name; describe more complex techniques in the Methods section.*
☐	☒	A description of all covariates tested
☐	☒	A description of any assumptions or corrections, such as tests of normality and adjustment for multiple comparisons
☐	☒	A full description of the statistical parameters including central tendency (e.g. means) or other basic estimates (e.g. regression coefficient) AND variation (e.g. standard deviation) or associated estimates of uncertainty (e.g. confidence intervals)
☐	☒	For null hypothesis testing, the test statistic (e.g. *F*, *t*, *r*) with confidence intervals, effect sizes, degrees of freedom and *P* value noted<br>*Give P values as exact values whenever suitable.*
☒	☐	For Bayesian analysis, information on the choice of priors and Markov chain Monte Carlo settings
☒	☐	For hierarchical and complex designs, identification of the appropriate level for tests and full reporting of outcomes
☐	☒	Estimates of effect sizes (e.g. Cohen's *d*, Pearson's *r*), indicating how they were calculated

Our web collection on statistics for biologists contains articles on many of the points above.

Software and code

Policy information about availability of computer code

Data collection	All datasets used in the current study are published previously and publicly available. Details of each dataset including the citation to its original study and accession number are provided in the manuscript. All datasets used in the current study are also curated and made available in the github repository, https://sydneybiox.github.io/CPOP
Data analysis	The software CPOP used for data analysis is made available in the github repository, https://github.com/SydneyBioX/CPOP.

For manuscripts utilizing custom algorithms or software that are central to the research but not yet described in published literature, software must be made available to editors and reviewers. We strongly encourage code deposition in a community repository (e.g. GitHub). See the Nature Portfolio guidelines for submitting code & software for further information.

Data

Policy information about availability of data

All manuscripts must include a data availability statement. This statement should provide the following information, where applicable:

- Accession codes, unique identifiers, or web links for publicly available datasets
- A description of any restrictions on data availability
- For clinical datasets or third party data, please ensure that the statement adheres to our policy

All datasets used in the current study are published previously and available publicly. Accession codes, unique identifiers, references as well as other summary statistics for each of all datasets are provided in the manuscript. All datasets generated and/or analysed during the current study are submitted to GEO.

Field-specific reporting

Please select the one below that is the best fit for your research. If you are not sure, read the appropriate sections before making your selection.

☒ Life sciences ☐ Behavioural & social sciences ☐ Ecological, evolutionary & environmental sciences

For a reference copy of the document with all sections, see nature.com/documents/nr-reporting-summary-flat.pdf

Life sciences study design

All studies must disclose on these points even when the disclosure is negative.

Sample size	No sample size calculation is require in this study. All sample size for data collected in the current study are reported in detail.
Data exclusions	Data exclusions was not applicable to this study.
Replication	Data evaluation is performed on three independent data collections across three diseases consisting of 16 datasets.
Randomization	There was no allocation in this study and thus randomization was not application.
Blinding	Blinding was not applicable to this study

Reporting for specific materials, systems and methods

We require information from authors about some types of materials, experimental systems and methods used in many studies. Here, indicate whether each material, system or method listed is relevant to your study. If you are not sure if a list item applies to your research, read the appropriate section before selecting a response.

Materials & experimental systems

n/a	Involved in the study	
☒	☐	Antibodies
☒	☐	Eukaryotic cell lines
☒	☐	Palaeontology and archaeology
☒	☐	Animals and other organisms
☐	☒	Human research participants
☐	☒	Clinical data
☒	☐	Dual use research of concern

Methods

n/a	Involved in the study	
☒	☐	ChIP-seq
☒	☐	Flow cytometry
☒	☐	MRI-based neuroimaging

Human research participants

Policy information about studies involving human research participants

Population characteristics	Tumor samples and clinical data from Australia were obtained from the Melanoma Institute Australia (MIA) Biospecimen Bank, a prospectively collected repository of fresh-frozen tumors accrued with written informed patient consent (ref #14 Jayawardana 2015).
Recruitment	Participation has been offered prospectively to essentially all adult patients attending MIA and its affiliated clinics since 1996.
Ethics oversight	Sydney South West Area Health Service institutional ethics review committee (Royal Prince Alfred Hospital Zone) Protocol No. X08-0155/HREC 08/RPAH/262, No. X11-0023/HREC 11/RPAH/32, and No. X07-0202/HREC/07/RPAH/30).

Note that full information on the approval of the study protocol must also be provided in the manuscript.

Clinical data

Policy information about clinical studies

All manuscripts should comply with the ICMJE guidelines for publication of clinical research and a completed CONSORT checklist must be included with all submissions.

Clinical trial registration	N/A
Study protocol	Prospective observational, non-interventional protocol.
Data collection	Multiple clinical settings affiliated with MIA, comprehensive recording of clinical events and follow-up to record outcomes such as relapse and survival.

Outcomes	Relapse-free survival and overall survival.
